# The combined role of dispersal and niche evolution in the diversification of Neotropical lizards

**DOI:** 10.1002/ece3.6091

**Published:** 2020-02-14

**Authors:** Yumi Sheu, Juan P. Zurano, Marco A. Ribeiro‐Junior, Teresa C. Ávila‐Pires, Miguel T. Rodrigues, Guarino R. Colli, Fernanda P. Werneck

**Affiliations:** ^1^ Programa de Pós‐Graduação em Genética Conservação e Biologia Evolutiva Instituto Nacional de Pesquisas do Amazônia Manaus Brasil; ^2^ Programa de Pós‐Graduação em Genética e Melhoramento Universidade Federal do Espírito Santo Espírito Santo Brasil; ^3^ Departamento de Sistemática e Ecologia Universidade Federal da Paraíba João Pessoa Brasil; ^4^ School of Zoology Tel Aviv University Tel Aviv Israel; ^5^ Coordenação de Zoologia Museu Paraense Emílio Goeldi Pará Brasil; ^6^ Departamento de Zoologia Instituto de Biociências Universidade de São Paulo São Paulo Brasil; ^7^ Departamento de Zoologia Instituto de Ciências Biológicas Universidade de Brasília Brasília Brasil; ^8^ Programa de Coleções Científicas Biológicas Coordenação de Biodiversidade Instituto Nacional de Pesquisas da Amazônia Manaus Brasil; ^9^ Department of Organismic and Evolutionary Biology Museum of Comparative Biology Harvard University Cambridge MA USA

**Keywords:** Amazonia, Cerrado, ecotone, *Kentropyx*, phyloclimatic modeling, South America, speciation

## Abstract

Ecological requirements and environmental conditions can influence diversification across temporal and spatial scales. Understanding the role of ecological niche evolution under phylogenetic contexts provides insights on speciation mechanisms and possible responses to future climatic change. Large‐scale phyloclimatic studies on the megadiverse Neotropics, where biomes with contrasting vegetation types occur in narrow contact, are rare. We integrate ecological and biogeographic data with phylogenetic comparative methods, to investigate the relative roles of biogeographic events and niche divergence and conservatism on the diversification of the lizard genus *Kentropyx* Spix, 1825 (Squamata: Teiidae), distributed in South American rainforests and savannas. Using five molecular markers, we estimated a dated species tree, which recovered three clades coincident with previously proposed species groups diverging during the mid‐Miocene. Biogeography reconstruction indicates a role of successive dispersal events from an ancestral range in the Brazilian Shield and western Amazonia. Ancestral reconstruction of climatic tolerances and niche overlap metrics indicates a trend of conservatism during the diversification of groups from the Amazon Basin and Guiana Shield, and a strong signal of niche divergence in the Brazilian Shield savannas. Our results suggest that climatic‐driven divergence at dynamic forest‐savanna borders might have resulted in adaptation to new environmental niches, promoting habitat shifts and shaping speciation patterns of Neotropical lizards. Dispersal and ecological divergence could have a more important role in Neotropical diversification than previously thought.

## Graphical abstract

We integrated ecological and molecular data to investigate the relative roles of biogeographic events and niche evolution on the diversification of a lizard genus distributed in Neotropical rainforests and savannas. Our results suggest that dispersal and ecological divergence could have a more important role in Neotropical diversification than previously thought.

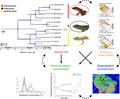

## 1 INTRODUCTION

Ecological requirements may have a fundamental role on speciation processes at multiple spatial and temporal scales, as natural selection may drive adaptive divergence of lineages from contrasting environments (Hua & Wiens, 2013; Rolland et al., 2018; Wang, Liu, et al., 2017; Wiens & Graham, 2005). Therefore, it is of utmost importance to comprehend the role of ecological niche evolution (here broadly defined by both niche divergence and stasis processes) in promoting diversification patterns, not only to understand how historical environmental change generated diversity but also to understand how speciation processes can be affected in the face of increasingly intense environmental and climatic changes. Such studies are especially necessary to support speciation research and conservation assessments in megadiverse and environmentally complex areas, such as the Neotropical region.

Niche evolution research has focused largely on evaluating signals of phylogenetic niche conservatism (PNC) and phylogenetic signal of variables that reflect ecological requirements. PNC is defined as the tendency of species to retain characteristics of their ancestral niche throughout time (Boucher, Thuiller, Davies, & Lavergne, 2014) and has been traditionally associated with allopatric speciation scenarios, in which the impossibility to track new niches created by geographical isolation would prevent gene flow and bolster speciation processes (Budic & Dormann, 2015; Wiens, 2004), favoring fixation of mutations by drift under similar environmental conditions (Schluter, 2009). However, niche conservatism may also cause ecological speciation when species follow changing favorable niches through time and slowly diverge from their ancestral niches (Pyron, Costa, Patten, & Burbrink, 2015). In this scenario, low‐to‐moderate levels of PNC would favor dispersal along changing environments and species divergence could result from differential adaptation to distinct ecological conditions, or the so‐called ecological speciation mechanisms (Schluter, 2009).

Phyloclimatic modeling emerged as new field of research that integrates ecological niche modeling (ENM) with dated phylogenies to explore the climatic factors influencing the geographic distribution of species (Evans, Smith, Flynn, & Donoghue, 2009; Jakob, Heibl, Rodder, & Blattner, 2010; Nyári & Reddy, 2013) and better understand how niche evolution and species divergence can relate. Phylogenetic comparative studies on niches of lineages inhabiting contrasting environments may provide valuable insights on the drivers of diversification along and across habitat transitions and on the future of species in global climate change scenarios (D'Amen, Zimmerman, & Pearman, 2013; Hoffmann & Sgro, 2011).

Broadscale phyloclimatic studies of Neotropical organisms are incipient (Seeholzer, Claramunt, & Brumfield, 2017; Zurano, Martinez, Canto‐Hernandez, Montoya‐Burgos, & Costa, 2017). Further, the effects of different components of the complex Neotropical landscape on taxa with different ecological tolerances are still unexplored in evolutionary contexts. The lizard genus *Kentropyx* Spix, 1825 (Squamata: Teiidae) is widely distributed in the contrasting environments of the wetter Amazon and Atlantic rainforests (*calcarata* species group) and in the drier open biomes of the South America dry diagonal‐Cerrado and Pantanal (*paulensis* species group) or the Amazon savannas (*striata* species group; Ribeiro‐Junior & Amaral, 2016a; Werneck, Giugliano, Collevatti, & Colli, 2009), which are important biodiversity centers in the Neotropical region (Antonelli, Ariza, et al., 2018). In general, Neotropical regions and biomes have changed considerably over time (Jaramillo & Cárdenas, 2013), making it important to consider historical changes in their connectivity and consequent impacts on biotic distribution and interchange. A broad biogeographic meta‐analyses combining distribution data and phylogenies for several groups (including Squamata) detected a high proportion of biotic interchange among Neotropical regions, mostly associated with dispersal shifts from the forest into the open biomes (Antonelli, Zizka, et al., 2018). Therefore, an integration between biogeographic and phyloclimatic approaches would be ideal to address whether biome shifts and niche evolution had determinant roles on the diversification of *Kentropyx*.

Initial studies proposed that during the evolution of *Kentropyx* from a hypothetic forest ancestor, dorsal scales enlarged in size (and conversely decreased in number) as thermal and hydric adaptations to drought (i.e., larger dorsal scales have less surface contact area and lower evaporation and thermoregulation rates; Horton, 1972), associated with occupation of open environments (Gallagher & Dixon, 1992; Gallagher, Dixon, & Schmidly, 1986), with species from the *calcarata*, *paulensis*, and *striata* groups successively diverging. However, these studies did not use phylogenetic methods and the monophyly of the species groups was still uncertain. Since then, the evolutionary history of *Kentropyx* has been addressed and the monophyly of the three species group confirmed (Werneck et al., 2009), but their phylogenetic position and biogeographical history remain unclear. For example, it is unknown whether the ancestral range of the genus was centered on forested or open regions and if speciation and morphological divergence were associated with neutral genetic drift or ecologically driven adaptive scenarios (Werneck et al., 2009). In this context, *Kentropyx* is an ideal model system to investigate the interplay between niche evolution and allopatric biogeography processes in promoting speciation at contrasting environments subject to different climatic and landscape histories in the megadiverse Neotropics.

Here, we implement a phyloclimatic approach integrating refined historical and ecological inferences to assess the relative roles of biogeographical events and climatic niche evolution on the speciation and evolutionary history of this group of Neotropical lizards. We first infer the phylogenetic relationships, divergence times, and biogeographic history of *Kentropyx* through a species tree and reconstructions inferred from multiple markers and dense intraspecific sampling for most species of the genus. We then use an extensive geographic dataset for all species as the basis for the interpretation and analysis of climatic niche evolution of the genus. If allopatric speciation due to vicariant processes and geographic isolation was a preponderant process, we expect the cladogenesis pattern to coincide spatially and temporally with large‐scale Neotropical geomorphological events and to recover PNC (or large niche overlap) across most species‐pairs comparisons, even when geographic overlap is low (including more distantly related species). On the other hand, if ecological speciation was a preponderant process, we expect to find evidence for dispersal processes acting on biogeographic reconstructions and lower signals of PNC, causing niche divergence as species follow changing favorable niches through time. Specifically, for the biological *Kentropyx* system, we expect that species from the same species group will present greater similarity of ecological niches than comparisons between species from different species groups, indicating that niche conservatism may have been important in their speciation. On the other hand, we expect greater niche differences between species from different species groups, indicating that niche divergence had a greater role in speciation in these cases.

## 2 MATERIAL AND METHODS

### 2.1 Target taxa: *Kentropyx* taxonomic context

*Kentropyx* differs from other teiid genera by the presence of strongly keeled ventral scales, being considered the most morphologically distinct genus of the Teiinae (Gallagher & Dixon, 1992; Harvey, Ugueto, & Gutberlet, 2012). According to Harvey et al. (2012), there are nine known species classified into three species groups based on dorsal scale characteristics.

The *calcarata* group (*Kentropyx calcarata*, *Kentropyx pelviceps*, and *Kentropyx altamazonica*) is characterized by small and granular dorsal and lateral scales, clearly distinct from the keeled, plate‐like supracaudal scales, and occurs in forest formations in Amazonia, the Guiana Shield, and the Atlantic Forest (Ribeiro‐Junior & Amaral, 2016a). Based on meristic characters, Costa (2015) proposed the revalidation of *Kentropyx vittata* for the Atlantic Forest taxon distributed south of the São Francisco river, which is currently synonymized under *K. calcarata*. However, since this proposal has not been published and the species limits were not investigated with genetic data, we here refer to all *K. calcarata* samples disjunctly distributed in the Atlantic Forest as a lineage within the recognized taxon (i.e., Atlantic Forest populations of *K. calcarata*; Franzini, Teixeira, Tavares‐Bastos, & Mesquita, 2019).

The *paulensis* group (*Kentropyx paulensis*, *Kentropyx viridistriga*, *Kentropyx vanzoi*, and *Kentropyx lagartija*), with granular dorsal and lateral scales that gradually increase in size toward the tail, where they cannot be distinguished easily from the supracaudals, occurs in open vegetation formations from Argentina, Bolivia, Paraguay, and Brazil (Werneck, 2011). Some consider *K. lagartija* as a synonym of *K. viridistriga* (Gallagher & Dixon, 1992), and Werneck et al. (2009) recognized a still undescribed species of *Kentropyx* from the Jalapão region in the state of Tocantins (Central Brazil) as part of this group. Here, we also recognize, based on morphological distinctiveness, another undescribed species from the *K. paulensis* group, from the region of Parque Nacional das Sempre‐Vivas in Diamantina, state of Minas Gerais (central Brazil).

Lastly, the *striata* group includes *Kentropyx striata*, associated with open formations from the Guiana Shield, southern margin of the Amazon River, and eastern nonforested Marajó Island (Ribeiro‐Junior & Amaral, 2016a) is characterized by plate‐like dorsal scales in longitudinal rows and lateral granular scales (Gallagher & Dixon, 1992; Werneck et al., 2009). Initially, *Kentropyx borckiana*, from northern South America and without known records for Brazil, was proposed to be part of the *striata* group (Gallagher & Dixon, 1992). Then, molecular evidence confirmed that *K. borckiana* is parthenogenetic, with a possible hybrid origin between *K. calcarata* and *K. striata* (Cole, Dessauer, Townsend, & Arnold, 1995; Reeder, Cole, & Dessauer, 2002).

### 2.2 Sampling, sequence data collection, and data preparation

**Figure 1:**
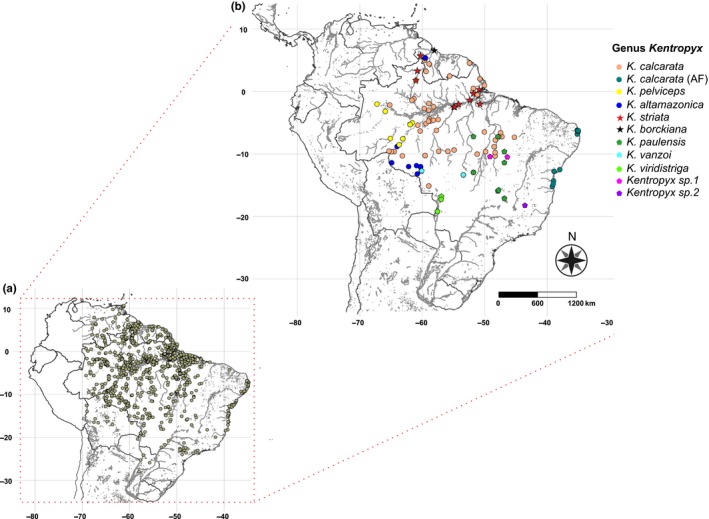
Distribution of sampling localities for *Kentropyx* lizards. (a) Occurrence points used to estimate niche models depicted as the complete dataset for the genus; (b) samples used to generate sequence data, identified by colored symbols: circles (*calcarata* species group; AF stands for the Atlantic Forest populations of *Kentropyx calcarata*), stars (*striata* species groups), and pentagon (*paulensis* species group)

We collected molecular data from tissue samples (muscle or liver) of 180 specimens, representing 11 species of *Kentropyx* (include all valid species except for *K. lagartija*, plus two undescribed species from the *K. paulensis* group and one from the *K. calcarata* group) from 73 distinct localities (Figure 1b and Table S1; Supporting Information deposited in the Dryad Digital Repository: https://doi.org/10.5061/dryad.zkh18936n). This is the most comprehensive sampling ever implemented for the genus, both at interspecific and intraspecific levels. We used *Ameiva ameiva* as an out‐group for phylogenetic analysis (Table S1). All laboratory procedures were carried out at the Laboratório Temático de Biologia Molecular from the Instituto Nacional de Pesquisas da Amazônia (LTBM/INPA).

The Wizard Genomic DNA Purification Kit (Promega) was used for DNA extraction, and fragments of mitochondrial and nuclear genes were amplified through the polymerase chain reaction (PCR) using a GoTaq Green MasterMix (Promega). We used two mitochondrial genes, cytochrome b (cyt *b*) and ribosomal RNA 16S, and four nuclear genes, RP40, R35, SNCAIP, and DNH3. Details for primers and protocols used are listed in Table S2. PCR purification was done through the addition of polyethylene glicol (PEG, 1 g/ml). Sequencing reactions were done using the DYEnamicTM ET terminator cycle sequencing kit (Amersham Pharmacia Biotech) on an ABI3130xl sequencer. Sequences obtained were edited with Geneious 7.0 (Kearse et al., 2012) and aligned with Muscle (Edgar, 2004). Evolutionary models for phylogenetic reconstruction were estimated for each gene using the Bayesian information criterion (BIC) in JModeltest 2.1.7 (Darriba, Taboada, Doallo, & Posada, 2012), while the gametic phases for nuclear markers of heterozygous individuals were resolved with PHASE 2.1.1 (Stephens, Smith, & Donnelly, 2001). We deposited all sequences in GenBank (accession nos. cyt *b*: MN873067—MN873164, AM343; SNCAIP: MN873165—MN873345, MN879736, AM343; DNH3: MN873346—MN873526; R35: MN877443—MN877625; RP40: MN925873—MN926046). Detailed accession numbers per sample and marker are available at Table S3.

### 2.3 Phylogenetic analyses and divergence dating

We estimated gene trees for each marker and concatenated sequences using Bayesian inference under a Monte Carlo chain (MCMC) with 10,000,000 generations (trees sampled every 1,000th generation) in MrBayes 3.1.2 (Ronquist & Huelsenbeck, 2003). We used TRACER 1.6.0 (Rambaut, Suchard, & Drummond, 2014) to evaluate convergence to stationarity for all sampled parameters, discarding the initial 10% of generated trees as burn‐in.

We estimated a species tree from the multilocus gene trees under a coalescent model and simultaneously estimated divergence times using *BEAST 1.8.2 (Drummond & Rambaut, 2007; Heled & Drummond, 2010). Divergence estimates were calibrated based on a mtDNA substitution rate of 0.65% changes/million years, commonly used to date squamate phylogenies (Macey et al., 1998), and nuclear loci rates were estimated from the data using the mtDNA rate as a reference, following an uncorrelated lognormal relaxed clock and a Yule speciation prior (Yule Process, pure‐birth). We implemented five independent runs of 200,000,000 generations each, sampling every 20,000 generations. Runs and trees were combined with LogCombiner (Drummond & Rambaut, 2007) discarding the initial 10% of the posterior samples as burn‐in. We computed the maximum clade credibility (MCC) tree based on all loci with TreeAnnotator v1.4.8 and traced and visualized gene trees and species trees with FigTree v1.3.1 (Drummond & Rambaut, 2007).

### 2.4 Biogeographic reconstruction

We identified seven main geographic areas inhabited by *Kentropyx*: forest formations from the Guiana Shield (FGS), open formations from the Guiana Shield (OGS), west Amazon Basin (WAB), east Amazon Basin (EAB), Brazilian Shield (BS), Atlantic Forest (AF), and Chaco‐Paraná Basin (CPB). Then, we used a probabilistic approach (in a likelihood framework) to model the evolution of the geographic distribution of *Kentropyx* lizards based on the estimated species tree and using the BioGeoBEARS package (Matzke, 2014, 2015) in R v.3.2.5 (R Core Team, 2019). BioGeoBEARS allows running the analyses with different models of geographic range evolution and compares their likelihood by estimating the probability of the data under each model. All models were tested, including dispersal–extinction–cladogenesis (DEC; Ree & Smith, 2008), geographic Bayesian inference (BAYAREA; Landis, Matzke, Moore, & Huelsenbeck, 2013), and dispersal‐vicariance (DIVALIKE; Ronquist, 1997). In addition, we tested the speciation parameter with the founder effect, jump dispersion (+J), for all models. Model assessment was based on the Akaike information criterion (AIC).

### 2.5 Distribution records and ecological niche modeling

We compiled distribution records from recent revisions in which identifications and occurrences were carefully verified based on vouchers deposited in several scientific collections (Ribeiro‐Junior & Amaral, 2016a, 2016b). The dataset was completed with additional occurrence data from the literature (Harvey et al., 2012; Werneck et al., 2009), from records compiled during the process of assembly of the list of Brazilian lizard species organized by the ICMBio governmental agency (GRC, Unpublished Data), and online databases (http://splink.cria.org.br). We also included occurrence data for the samples we sequenced, for a total of 1,595 distribution records for the eleven species represented in the species tree (Figure 1; Table S4). To address problems associated with spatial sampling biases, we used a 5‐km distance ratio for spatial filtering of occurrence records using the R packages spThin v. 0.1.0.1 (Aiello‐Lammens, Boria, Radosavljevic, Vilela, & Anderson, 2019). To do so, we sample the unfiltered dataset 100 times and then randomly select one of the datasets that produced the maximum number of occurrence localities remaining (Aiello‐Lammens, Boria, Radosavljevic, Vilela, & Anderson, 2015). Total number of records per species before and after spatial filtering were, respectively, *K. calcarata* (785/519), *K. calcarata*_AF (68/50), *K. altamazonica* (255/223)*, K. pelviceps* (209/185)*, K. paulensis* (50/46), *K. vanzoi* (27/25), *K. viridistriga* (20/17), *Kentropyx* sp1 from Tocantins (13/3), *Kentropyx* sp2 from Minas Gerais (1/1), *K. striata* (152/119), and *K. borckiana* (15/9). The low number of records available for *Kentropyx* sp. 1 e *Kentropyx* sp. 2 is due to the fact that they are not formally described and were only recently recognized (Werneck et al., 2009), so their actual ranges are still unknown. As such, we opted not to implement our niche analysis for these two species. However, we used both species on the species tree reconstruction and removed them a posteriori from the inferred tree.

We used 19 bioclimatic variables (http://www.worldclim.org/com) at a spatial resolution of 2.5 arcmin (~5 km) that summarized current precipitation and temperature data (Hijmans, Cameron, Parra, Jones, & Jarvis, 2005), one global aridity index variable (http://www.csi.cgiar.org), and one vegetation cover variable (https://globalmaps.github.io/ptc.html). We tested for correlation between variables using a Pearson correlation matrix (*r* > .8) to avoid overparameterization of our ENMs with redundant climatic variables (Phillips, Anderson, & Schapire, 2006). The complete list of all 12 variables used in the downstream analysis is available in Table S5.

We built the ENMs using Maxent v.3.4.1 (Phillips et al., 2006) with ENMeval v.0.3.0 package (Muscarella et al., 2014) in R 3.6.0 (R Core Team, 2019). After partitioning occurrence data using the checkerboard1 method (Muscarella et al., 2014), we built models with regularization multiplier values ranging from 0.5 to 4.0 (increments of 0.5) and with six different feature class combinations (L, LQ, H, LQH, LQHP, LQHPT; where L = linear, Q = quadratic, H = hinge, P = product and T = threshold); this resulted in 48 models for each species. Among candidate models for each species, we selected the model with lowest delta value of corrected Akaike's information criterion for small sample sizes (AICc), which reflects both models goodness of fit and complexity (Muscarella et al., 2014). The selected models (detailed at Table S5) were used for the downstream analyses of ancestral niche reconstruction. To evaluate model performance, we also report the area under the curve (AUC) of the receiver operating characteristic plot, a threshold‐independent measure of model performance as compared to null expectations, based on the testing data (AUC_TEST_), and the difference between training and testing AUC (AUC_DIFF_). AUC_DIFF_ provides a quantification of overfitting and is expected to be positively associated with the degree of model overfitting (Muscarella et al., 2014).

### 2.6 Ancestral niche reconstruction

Recent methods have made possible not only to characterize but also to quantify the amplitude of bioclimatic variables in occupancy of niche during species diversification (Evans et al., 2009; Jakob et al., 2010; Nyári & Reddy, 2013). Predicted niche occupation (PNO) profiles were obtained from ENMs and correspond to the tolerances or occupation profiles of the climatic dimensions by each species for each bioclimatic variable. To generate PNOs, each bioclimatic layer used in the ecological niche modeling algorithm is integrated with each MaxEnt species distribution probability, resulting in suitability predictions for each species in the occupation of each bioclimatic variable. We then evaluated niche evolution through ancestral niche reconstruction analyses for each PNO, estimating the maximum‐likelihood value for each climatic variable in the internal nodes of the calibrated phylogeny inferred by our study, assuming a Brownian model of evolution (Felsenstein, 1985, 1988). For both analyses (PNO and ancestral niche reconstruction), we used Phyloclim v.9.5 package (Heibl & Clement, 2015) implemented in R v.3.6 (R Core Team, 2019). We recognize that macroclimatic parameters do not necessarily reflect the microclimatic conditions faced by these lizards, but we regard them adequate to infer environmental niche shift across lineages.

### 2.7 Niche conservatism and divergence comparisons

To analyze patterns of niche conservatism and divergence among all species pairs of *Kentropyx*, we used the same environmental variables used to build ENMs and the PCA‐env framework of Broennimann et al. (2012). This approach corrects for potential sampling bias by dividing the number of times that the species occur in an environment by the frequency of localities in the region that have those environmental conditions. In addition to the georeferenced records observed, to characterize the background of environmental conditions for each species, we used the ecoregions in which each species of *Kentropyx* occurs according to the global biogeographic classification proposed by Olson et al. (2001). The PCA was calibrated using environmental values from all the pixels of the species being compared. Then, the PCA scores of the two species distributions for which the niches are under comparison were projected onto a grid of cells bounded by the minimum and maximum PCA scores in the study areas. A smoothed density of occurrences for each species in each cell of the grid is then estimated using a kernel density function (Broennimann, Di Cola, & Guisan, 2018; Di Cola et al., 2017). Grid densities were then used to calculate an observed niche overlap score of each species pair using Schoener's *D* metric (Schoener, 1968), ranging from 0 to 1 (0 indicating no overlap/complete divergence and 1 indicating complete/high overlap; Warren, Glor, & Turelli, 2008). Finally, to test the conservatism or divergence niche hypotheses, we compared whether the observed niche overlap value for each pair of species was more similar (niche conservatism) or more different (niche divergence) than the niche overlap expected by chance. If the observed value of *D* is outside the 95% confidence interval of the random niche overlap values generated with 1,000 simulations, we accepted the conservatism (if above) or divergence (if below) of niches. To do so, we use the niche similarity test available in Ecospat v.3.0 packages with 1,000 replications (Broennimann et al., 2018) in R 3.6 (R Core Team, 2019). The niche similarity test assesses, through random shifts of the niches within available conditions in the study area, whether the species' niches are more or less similar than expected by chance (Di Cola et al., 2017).

## 3 RESULTS

### 3.1 Phylogenetic relationships, dating, and biogeographic scenario

**Figure 2:**
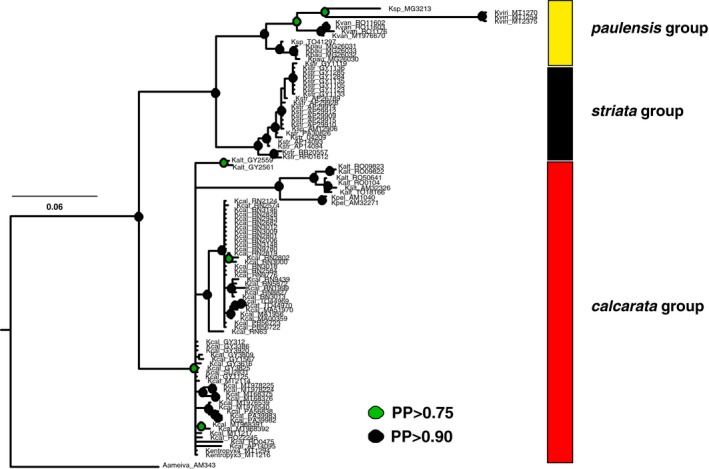
Mitochondrial (cyt *b*) gene tree estimated by Bayesian inference. Green and black circles indicate nodes with posterior probabilities higher than 75% and 90%, respectively. The three species groups are recovered: *calcarata* (red bar), *paulensis* (yellow bar), and *striata* (black bar)

We sequenced a total of 1,058 base pairs (bp) for mitochondrial genes and 1,946 bp for nuclear genes. The list of markers and their estimates of nucleotide and haplotype diversity is available at Table S6. Among all gene trees, the cyt *b* gene tree showed a stronger structure for *Kentropyx*, recovering the three species groups (Figure 2). The forest clade composed by *K. altamazonica*, *K. calcarata*, Atlantic Forest populations of *K. calcarata*, and *K. pelviceps* (i.e., *calcarata* group), is sister to a clade of open vegetation species that includes two subclades, one formed by *K. vanzoi*, *K. paulensis*, *Kentropyx* sp. 1, *Kentropyx* sp. 2, and *K. viridistriga* (i.e., *paulensis* group) and the other by *K. striata* (i.e., *striata* group). The cyt *b* gene tree also recovered considerable intraspecific structure for some of the species (Figure 2). In contrast, some of the nuclear gene trees had little resolution, presenting in some cases paraphyly for some species and many polytomies (Figures S1 and S2). The 16S mitochondrial gene evidenced high differentiation among sequences without, however, presenting interspecific resolution, indicating saturation generating uncertainties regarding the phylogenetic relationships. Thus, we chose not to use 16S for subsequent species tree and divergence time estimate analyses. Concatenated nuclear genes resulted in a better‐resolved topology than individual gene trees. However, there was limited interspecific resolution, especially for species of the *calcarata* group (Figure S1).

**Figure 3:**
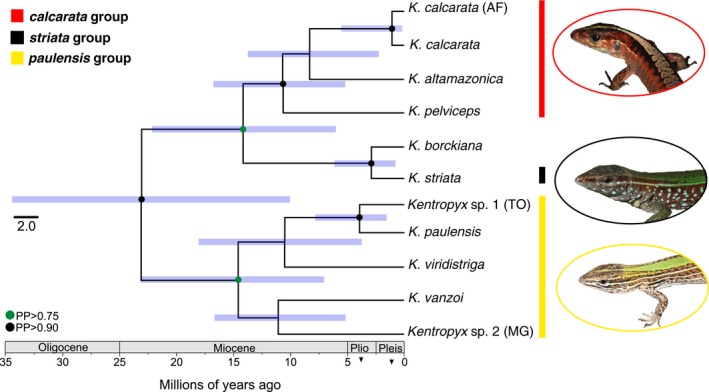
*Kentropyx* species tree (maximum clade credibility tree based on five loci) and divergence time estimates, derived under a coalescent model with ∗BEAST. Blue bars represent the credibility intervals, and vertical colored bars depict the species groups as in Figure 2. Green and black circles indicate nodes with posterior probabilities higher than 75% and 95%, respectively. Photographs representing one species from each of the species group: *Kentropyx pelviceps* (photo by Miguel T. Rodrigues); *Kentropyx striata* (photo by Miguel T. Rodrigues), and *Kentropyx viridistriga* (photo by Helen Pheasey)

The multilocus species tree inferred with *BEAST recovered with low (*paulensis*) and high (*calcarata* and *striata*) support the monophyly of the three species groups of *Kentropyx* (Figure 3). The tree shows an initial divergence (PP = 0.99) between the *paulensis* group (from the diagonal of open formations) and the common ancestor of the *calcarata* (from the tropical rainforests) and *striata* (from the Amazonian open formations) groups during the early Miocene, about 23 million years ago—Mya (Figure 3). The divergence between the *calcarata* and *striata* groups was estimated at approximately 15.55 Mya during the middle Miocene, with *K. pelviceps* (PP = 0.93) the first to diverge within the *calcarata* group, followed by the split between *K. altamazonica* (PP = 0.65) and *K. calcarata*. The divergence between the Amazonian and the Atlantic forest lineages of *K. calcarata* dates from the middle Pleistocene (PP = 1), while *K. borckiana* diversification (due to possible hybridization between *K. striata* and *K. calcarata*) started approximately 3.02 Mya during the late Pliocene (PP = 0.99). The initial event responsible for the divergence of species of the *paulensis* group (PP = 0.77) occurred in the middle Miocene about 15.85 Mya. Within the *paulensis* group, *Kentropyx* sp. from Minas Gerais and *K. vanzoi* were the first to diverge at 12.79 Mya ago, followed by *K. viridistriga* which is sister of the clade including *Kentropyx* sp. from Tocantins and *K. paulensis* (PP = 0.90, Figure 3) the latter two diverging in the Pliocene.

**Figure 4:**
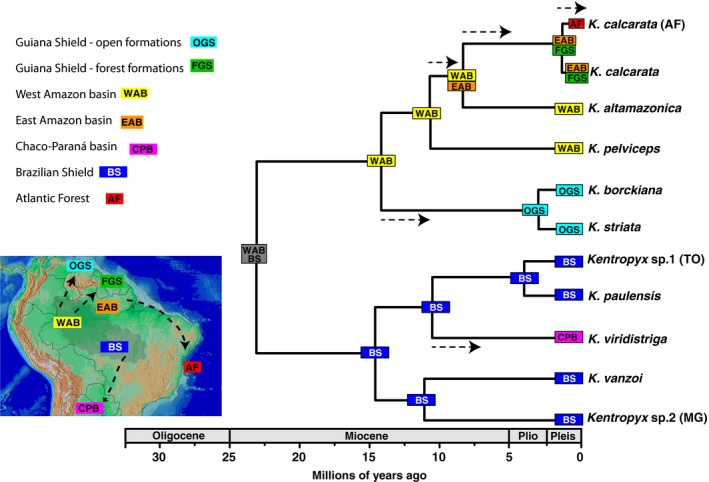
Biogeographic scenario inferred for *Kentropyx* by the best fit model recovered by BioGeoBEARS (DEC+J). Main biogeographical areas defined based on *Kentropyx* distribution are shown on the map as follows: Guiana Shield—open formations (light blue), Guiana Shield—forest formations (green), Western Amazonia (yellow), Eastern Amazonia (orange), Chaco‐Paraná basin (pink), Brazilian Shield (blue), and Atlantic Forest (red). The shaded gray area represents the ancestral distribution recovered for the genus, and arrows at the phylogeny and the map represent the dispersal events inferred to have happened along the branches. See Figure 3 for clades' posterior probabilities

Biogeographic reconstruction with BioGeoBEARS selected the dispersal–extinction–cladogenesis model with founding speciation event (DEC+J) as the best, followed by the DIVA+J model (Figure 4, Figure S3). The ancestral distributions reconstructed by the DEC+J model yielded LnL = −23.22 and AIC = 52.45, while the DIVALIKE+J reconstructions yielded LnL = −24.41 and AIC = 54.82 (Table 1). In general, all models implemented with the founder event (J) were better than the same models without this parameter. The best fit DEC+J model suggested that the origin of the genus occurred from an ancestor distributed in the Brazilian Shield that underwent a dispersal event before forming a large Amazonian lineage (*calcarata* + *striata* groups), first occupying the western part of the Amazon basin and subsequently open areas in the Guiana Shield at about 15.5 Mya, where *K. striata* diversified during the Pliocene–Pleistocene transition (Figure 4). In addition, diversification of the *calcarata* group culminated in a secondary colonization of eastern Amazonia approximately 8.8 Mya and the final establishment of *K. calcarata* populations in the Atlantic Forest by a subsequent founding dispersal (Figure 4). Concomitantly, significant diversification occurred in the Brazilian Shield, forming species of the *paulensis* group, with a secondary dispersion of *K. viridistriga* ancestor to the Chaco‐Paraná Basin (Figure 4).

**Table 1:** Likelihood estimates and parameters inferred by the biogeographic models tested with BioGeoBEARS

Models	LnL	Number of parameters	*d*	*e*	*j*	AIC
DEC	−28.63	2	0.011	1.47e−12	0	61.27
**^++^DEC+J**	**−23.22**	**3**	**0.004**	**1.0e−12**	**0.061**	**52.45**
DIVALIKE	−26.04	2	0.009	1.0e−12	0	56.53
^+^ **DIVALIKE+J**	**−24.41**	**3**	**0.003**	**1.0e−12**	**0.079**	**54.82**
BAYAREALIKE	−32.84	2	0.021	6.70e−12	0	69.69
BAYAREALIKE+J	−25.08	3	0.002	6.91e−12	0.101	56.17

Note

Best model (++) and second‐best model (+) are in bold.

Abbreviations: *d*, dispersion rate along the branches per million years; *e*, extinction rate along the branches per million years; *j*, founder‐event speciation, weighted by speciation event; LnL, log likelihood.

### 3.2 Ecological niche characterization

**Figure 5:**
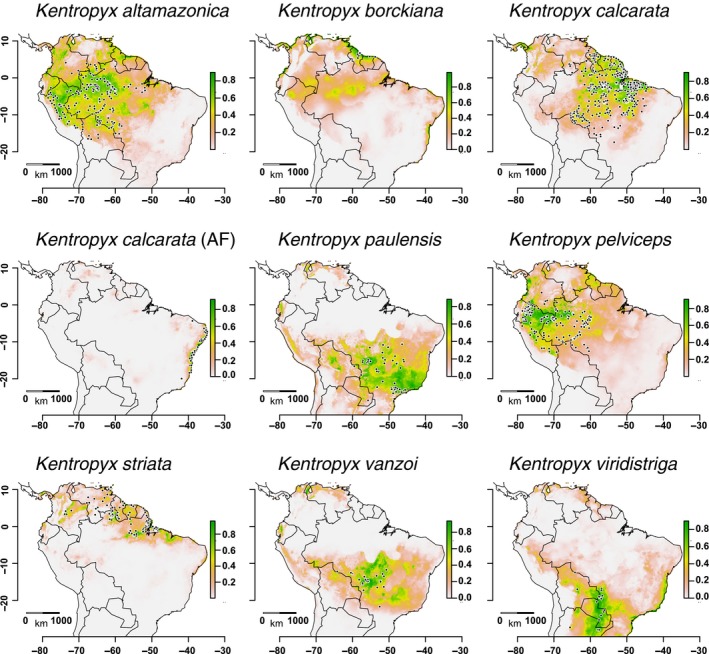
Ecological niche models estimated for the nine species of *Kentropyx*. Occurrence points of each species are plotted with black circles

Estimated ENMs illustrate well the known distributions and great heterogeneity of habitats occupied by *Kentropyx* species (Figure 5). Models did not over predict the known species distributions, except for *K. vanzoi* and *K. viridistriga* that are not known to occur in predicted areas of high suitability in southeastern Brazil and the Brazilian Atlantic coast, respectively. Among candidate models of each species, we selected the best performing model by AICc. All selected models have high AUC_TEST_ values, ranging from 0.84 to 0.98 (Table S5), indicating high performance of our predictions. Additionally, low values of AUC_DIFF_ (the difference between training and testing AUC) support that our models had low overfit (Muscarella et al., 2014; Warren & Seifert, 2011). The individual contribution of environmental predictors to ENMs reached up to 52.3% (Bio4; Table S5) and highlights that temperature seasonality and precipitation of driest quarter (Bio17) generally contributed more to the models in almost all species. *Kentropyx calcarata* is the species with the broadest distribution and occupying the most varied environments, such as the Guiana Shield, *terra firme* rain forest of Amazonia (western and eastern), Cerrado gallery forests, and the disjunct Atlantic Forest populations. *Kentropyx altamazonica* also occupies a wide range, mainly in western Amazonia and floodplain forests of eastern Amazonia, and *K. pelviceps* encompasses the *terra firme* rainforests of western Amazonia. *Kentropyx paulensis* and *K. vanzoi* share most of their distributions in the Central Brazilian Shield, and *K. viridistriga* occurs only in the Chaco‐Paraná Basin. *Kentropyx vanzoi*, in the southwestern Brazilian Cerrado, and *K. striata* and *K. borckiana*, in the Guiana Shield, especially in the open formations located north of South America and along some rivers, had the most restricted distributions (Figure 5).

### 3.3 Niche evolution: Ancestral reconstructions, conservatism and divergence comparisons

**Figure 6:**
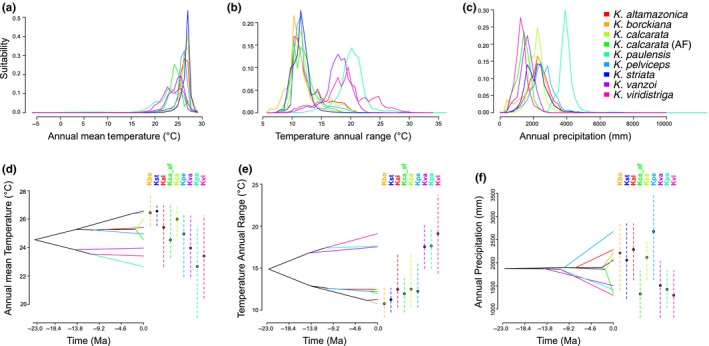
Ecological niche occupancy and evolution analyses for Neotropical *Kentropyx* lizards. (a–c) Predicted niche occupancy (PNO) profiles and (d–f) inferred history of the evolution of climatic tolerances for three selected variables (Bio1, Bio7, and Bio12) on the inferred calibrated species tree for nine *Kentropyx* species (as in Figure 3). The PNO horizontal axis represents the occupancy of annual mean temperature (Bio1), temperature annual range (Bio7), and annual precipitation (Bio12); and the vertical axis represents the suitability of each species for each variable presented. We integrate the phylogenetic hypothesis based on the estimated species tree, and PNO analyzes to reconstruct the mean of climatic tolerances for internal ancestral nodes with maximum likelihood based on 100 repetitions of the PNOs on the internal nodes. Points on the dashed vertical lines correspond to the mean of 80% central density of climatic tolerance for each extant species. Species abbreviations are as follow: *Kentropyx altamazonica* (Kalt), *Kentropyx borckiana* (Kbor), *Kentropyx calcarata* (Kcal), Atlantic Forest populations of *K. calcarata* (Kcal_AF), *Kentropyx paulensis* (Kpau), *Kentropyx pelviceps* (Kpel), *Kentropyx striata* (Kstr), *Kentropyx vanzoi* (Kvan), and *Kentropyx viridistriga* (Kviri)

From the models generated by Maxent, we inferred the PNO profiles and the evolutionary history of niche occupancy. PNOs showed a subtle heterogeneity in the occupation of each bioclimatic variable among the species groups of *Kentropyx*, especially in the contrast between the *paulensis* group and the other two species groups (*calcarata* + *striata*; Figure 6a–c, Figure S4). Also, the phylogenetic analysis of PNOs evidenced the radiation of some species in a variety of climatic conditions (Figure 6d–f, Figure S5).

In general, there are differences between the two large clades (see species tree), with members of each lineage (*calcarata* + *striata*) tending to ecologically resemble each other more closely than members of the other lineage (*paulensis*). In addition, some trends can be identified along the environmental gradient during the genus diversification. First, there is a tendency of increased annual average temperature (Bio1; Figure 6a,d) and annual precipitation (Bio12; Figure 6c,f) for the *calcarata* + *striata* clade and the reverse occurs for *paulensis* clade, which tends for lower temperatures and drier environments.

In contrast, there is a decrease in the temperature annual range (Bio7) for *calcarata* + *striata*, whereas the inverse occurs for *paulensis* clade (Figure 6b–e). Their common ancestor (*paulensis* group), at about 15 Mya, presented higher optimum values of temperature annual range, estimated as approximately 16°C (Figure 6e). Thus, it is possible to observe a large divergence in the climatic space between the species of the *paulensis* group (*K. paulensis*, *K. vanzoi*, and *K. viridistriga*) that occupy the Cerrado of the Brazilian Shield and the Chaco‐Paraná basin, and all other species. Other PNO profiles and historical reconstructions of climatic tolerances recovered a similar pattern of overlap of tolerance distributions between species groups, indicating that the *paulensis* group represents a potential case of ecological disparity within the genus (Figure S5). In general, most ancestral reconstructions showed that early in the evolutionary history of genus, there was a prominent increase in ecological divergence between groups (disparity between groups occurred) and a subsequent decrease in differences between groups, with the occupation of different niches by species (Figure S5).

For the PCA‐env analysis, we used the first two PCA axes that account for 52.12% and 17.77% of the total variance. The niche overlap between species pairs (*D* index) varied between 0 (e.g., *K. borckiana*—*K. paulensis* and *K. altamazonica*—*K. vanzoi*) and 0.54 (e.g., *K. altamazonica*—*K. pelviceps*; Table 2). Only three comparisons showed *D* values greater than 0.30, all involving species from the *calcarata* and *striata* groups (i.e., *K. borckiana*—*K. calcarata* [0.33], *K. calcarata*—*K. striata* [0.32], *K. altamazonica*—*K. pelviceps* [0.54]). On the other hand, the vast majority of the comparisons resulted in very low overlap values (Table 2) and significant support for niche divergence, particularly for pairwise comparisons involving species from the *paulensis* group (Table 3). According to Rödder and Engler (2011), values between 0 and 0.2 do not support overlap or indicate very limited overlap. Thus, in those cases where we did not observe niche overlap among species (*D* = 0), we supported niche divergence.

**Table 2:** Measures of niche overlap (*D*) for pairwise comparisons between *Kentropyx* species

	Kalt	Kcal	Kcal_AF	Kpel	Kbor	Kstr	Kpau	Kvan
Kcal	0.06							
Kcal_AF	0.01	0.15						
Kpel	0.54	0.02	0,02					
Kbor	0.10	0.33	0.04	0.02				
Kstr	0.2	0.32	0.05	0.02	0.08			
Kpau	0.01	0.02	0.06	0.01	0	0		
Kvan	0	0.02	0	0	0	0	0.04	
Kviri	0.01	0.01	0.09	0	0	0	0.08	0.01

Note

Species abbreviations are as follow: Kalt, *Kentropyx altamazonica*; Kbor, *Kentropyx borckiana*; Kcal, *Kentropyx calcarata*; Kcal_AF, Atlantic Forest populations of *K. calcarata*; Kpau, *Kentropyx paulensis*; Kpel, *Kentropyx pelviceps*; Kstr, *Kentropyx striata*; Kvan, *Kentropyx vanzoi*; and Kviri, *Kentropyx viridistriga*.

**Table 3:** Measures of similarity for pairwise comparisons between *Kentropyx* species (below diagonal **x → y**, above **y → x**)

	Kalt	Kcal	Kcal_AF	Kpel	Kbor	Kstr	Kpau	Kvan	Kviri
Kalt	—	0.47	0.47	0.07	0.08	0.46	0.45	**(D)**	0.61
Kcal	0.52	—	0.29	0.56	0.05	0.14	0.56	0.25	0.46
Kcal_AF	0.53	0.70	—	0.40	0.27	0.40	0.52	**(D)**	0.46
Kpel	0.92	0.41	0.61	—	0.23	0.44	0.64	0.63	0.64
Kbor	0.93	0.094	0.72	0.76	—	0.18	**(D)**	**(D)**	**(D)**
Kstr	0.55	0.85	0.61	0.51	0.82	—	**(D)**	**(D)**	**(D)**
Kpau	0.56	0.41	0.47	0.34	**(D)**	**(D)**	—	0.31	0.37
Kvan	**(D)**	0.73	**(D)**	0.38	**(D)**	**(D)**	0.66	—	0.55
Kviri	0.40	0.54	0.56	0.35	**(D)**	**(D)**	0.64	0.47	—

Note

Significant values at *p* < .05 for the niche similarity tests are in bold: conservatism (C), divergence (D). Species abbreviations are as follow: Kalt, *Kentropyx altamazonica*; Kbor, *Kentropyx borckiana*; Kcal, *Kentropyx calcarata*; Kcal_AF, Atlantic Forest populations of *K. calcarata*; Kpau, *Kentropyx paulensis*; Kpel, *Kentropyx pelviceps*; Kstr, *Kentropyx striata*; Kvan, *Kentropyx vanzoi*; and Kviri, *Kentropyx viridistriga*.

## 4 DISCUSSION

We present an integrative approach to understand the historical and ecological forces that drove the diversification of a lizard genus associated with open and forested formations in South America, exploring the evolution of the climatic niche occupancy in an explicit phylogenetic context. This integration sheds light over the complex biogeographical and evolutionary history of ecological tolerances, with possible adaptation to new niches, of this dynamic group of Neotropical lizards.

### 4.1 Systematics and historical biogeography

Phylogenetic tree reconstructions supported the monophyly of the previously recognized species groups of *Kentropyx* (Gallagher & Dixon, 1980, 1992), agreeing with earlier studies using morphology (Harvey et al., 2012), and morphology and mitochondrial data combined (Werneck et al., 2009). Conversely, these results are in contrast with a recent phylogenomic study of Teiidae (Tucker et al., 2016) in which some *Kentropyx* species were recovered as paraphyletic. However, those authors highlighted the need of additional sampling to properly assess the species‐level taxonomy (Tucker et al., 2016), which our current study did with a considerable broader interspecific and intraspecific sampling.

Regarding shallow (intraspecific) structure, individual and concatenated gene trees showed shared haplotypes and internested relationships between species of the *calcarata* group. Gene tree disagreement may come from incomplete lineage sorting for the markers used, or due to some introgression between species (Edwards, 2009; Maddison & Knowles, 2006). Our data corroborate the phylogenetic relationships recovered by Werneck et al. (2009) for the *paulensis* group, indicating *Kentropyx* sp. 1 from Tocantins as the sister species of *K. paulensis* followed by the sister species *K. viridistriga*, and also recovered *Kentropyx* sp. 2 from Minas Gerais (not sampled in that study) as the sister species of *K. vanzoi*. Such patterns highlight the complexity of interspecific boundaries and relationships within *Kentropyx* and reinforce the need for additional genomic studies based on extensive geographic and intraspecific sampling to resolve the boundaries and phylogenetic relationships of the genus, especially among the forest species that occur mainly in Amazonia.

In summary, previous systematics studies investigated the interspecific relationships of *Kentropyx* spp. without implementing coalescent phylogenetic analyses based on independent molecular markers or broad intraspecific sampling. All previous molecular studies for the genus had only a few specimens sampled per species, which hindered the detection of complex speciation patterns, such as evidence of introgression, hybridization, or incomplete lineage sorting among species. Our study contributes to a considerable increase in the sampling effort for the genus *Kentropyx*. We acknowledge potential limitations due to moderate support of a few nodes, suggesting that interspecific relationships of *Kentropyx* still deserve further investigation based on genomic approaches, especially to solve conflicts regarding species boundaries in the *calcarata* group.

Our biogeographical results differ in some respects from the scenario presented by Werneck et al. (2009) that recovered *K. striata* as the first species to diverge, being the sister taxon to all other species in the phylogeny. In our multilocus species tree, we found an early dichotomy that separates species that occur closer to the Equator, occupying both forest and open formations (i.e., *calcarata* group + *striata* group clade), from species that range in open areas in the Brazilian Shield (*paulensis* group). Thus, our results also differ from previous hypotheses which suggests that species diversification would have followed a linear progression of the condition of dorsal scales, with species from the *striata* group (with fewer and wider dorsal scales) supposedly diverging last as an adaptation to thermal and water regulation in dry environments (Gallagher & Dixon, 1992). As such, if the size and number of dorsal scales indeed had any adaptive significance within *Kentropyx* evolution, their differences would have evolved early on the group diversification.

The biogeographical scenario recovered implies that there was an initial divergence, during the Oligocene–Miocene transition, between the *calcarata* + *striata* clade and the *paulensis* group, and that both originated from an ancestor distributed at the Brazilian Shield and western Amazonia. After an initial divergence, these two lineages became restrict to different environments: forest formations in western Amazonia and open formations in the Brazilian Shield, respectively, which went through distinct diffusion histories. Although Neogene geomorphological events have been considered decisive in the vicariant speciation of several vertebrate groups in South America (Antonelli et al., 2010; Giugliano, Collevatti, & Colli, 2007; Werneck et al., 2009), our biogeographic reconstruction results are consistent with a scenario in which successive dispersal events, rather than vicariance only, played significant roles on the divergence of *Kentropyx* at the continental scale. As such, large‐scale landscape and drainage rearrangements due to the uplift of the Andes and climatic factors (Hoorn & Wesselingh, 2010) likely promoted conditions for dispersal along changing environments.

The mid‐late Miocene transition was marked by intense speciation, both between species groups (i.e., the split between the *calcarata* and *striata* groups) and within species groups (i.e., within *calcarata* and *paulensis* groups). Several geomorphological processes have been implied as determinant to generate vertebrate speciation patterns in the open diagonal biomes during this period, such as the central Brazilian Shield uplift during the end of Miocene (Giugliano, Nogueira, Valdujo, Collevatti, & Colli, 2013; Guarnizo et al., 2016; Werneck, Gamble, Colli, Rodrigues, & Sites, 2012), regional topographic reorganizations, establishment of plateaus and depressions, and formation of altitudinal gradients (Guarnizo et al., 2016; Werneck, 2011). Such events can help explain the diversification of the *paulensis* group. Furthermore, as discussed below based on ecological data, changes in environmental conditions were also important in promoting ecological divergence in the species of the *paulensis* group.

Climatic fluctuations and habitat shifts during the Pliocene–Pleistocene (Cheng et al., 2013) were likely determinant for the divergence of *K. striata* at the Guiana Shield savannas and of *K. calcarata* populations at the Atlantic Forest, both with current patchy or disjunct distributions resulting from the colonization of founders with high dispersal capabilities. Such fluctuations and resulting vegetation changes may also have favored secondary contact and hybridization between *K. striata* and *K. calcarata*, the supposed parent species of *K. borckiana* (Cole et al., 1995; Reeder et al., 2002). A mid‐Pleistocene rainforest corridor is known to have enabled synchronous invasions of the Atlantic Forest by Amazonian lineages of other lizard species (Prates, Rivera, Rodrigues, & Carnaval, 2016; Prates, Xue, et al., 2016) and is congruent with an invasion of *K. calcarata* from eastern Amazon. On the other hand, intercalated savanna expansions might have facilitated emergence and fast isolation of *K. striata* in disjunct populations. It is interesting to note that *K. striata* demonstrated a noticeable intraspecific genealogical structure (Figure 2), despite its relative recent diversification in the phylogeny (Figure 3). Avila‐Pires, Palheta, Silva, and Sturaro (2017) observed that the geographic variation in *K. striata* morphology is not enough for subspecies or additional species recognition, also indicating a relatively recent separation between isolated populations. Available biogeographical and geomorphological evidence indicate that during drier glacial periods, forests might have contracted (Haffer, 1969; Prance, 1982), forcing transitions in plant and animal populations and fragmentation and recolonization processes at the large scale (Dutech, Maggia, Tardy, Joly, & Jarne, 2003; Flanagan et al., 2004; Scotti‐Saintagne et al., 2013). Furthermore, it was suggested that South American savannas had reached their maximum extent during the LGM, connecting savannah blocks located north (Guiana Shield) and south (in central Brazil Cerrado) of the Amazon River (Webb, 1991). However, palynological (Colinvaux, de Oliveira, & Bush, 2000), speleothems (Wang, Edwards, et al., 2017), climate modeling (Mayle, 2004), and biome's paleodistribution modeling (Ledo & Colli, 2017; Werneck, 2011) studies agree that, despite changes in temperature and humidity patterns, most of the central portion of the Amazonia remained forested through the LGM and potential savanna connections were not necessarily simultaneous (Bush, 2017). Recently, Barthe et al. (2017) tested different demographic scenarios of Quaternary population contraction and expansion of Guiana Shield forest tree species and recovered expansion signals for some species and contraction for others, merging the two hypotheses within the Quaternary climatic and geological disturbances. Additional population studies within *K. striata* and *K. calcarata* can contribute to better distinguish between biogeographic, demographic, and adaptive/ecological signatures of these species with either disjunct (at Atlantic Forest) or restricted (Guiana Shield savannas) distributions, and aid in explaining the hybridization event with *K. calcarata* at rainforest‐open formation ecotonal regions of northern South America (Cole et al., 1995; Reeder et al., 2002).

### 4.2 The role of niche conservatism and divergence

*Kentropyx* species have occupied a great variety of environments in South America, from hot and humid areas of the Guiana Shield and the Amazon basin, to hot and seasonally dry areas of the Brazilian Shield. Niche evolution analyses show a clear divergence in the ecological space during the species group's separation. The optimal temperatures for species occurrence from the *calcarata* and *striata* groups, distributed in the north of South America, are on average 2°C hotter than for the species of the *paulensis* group in the Cerrado. Rainfall shows a similar pattern, with species from the *striata* and *calcarata* groups occupying areas of high precipitation in Amazonia promoted by high local evapotranspiration (Nobre, Obregón, Marengo, Fu, & Poveda, 2009), and *K. calcarata* lineages from the Atlantic forest experiencing slightly lower annual precipitation. The *paulensis* group occupies regions of the Cerrado with lower annual rainfall and two well‐defined seasons: dry (May to September) and rainy (October–April; Eiten, 1972).

Overall, most ancestral reconstructions based on PNOs' climatic tolerances and niche overlap/similarity comparisons demonstrated a similar dichotomy in occupation of niches between the sister clades distributed in northern South America (*calcarata* + *striata* groups) and in the Cerrado‐*paulensis* group (Figure 5a–c, Figure S4). This trend suggests that ancestors of the *paulensis* group occupied a very distinct ecological niche space since early the genus diversification, indicating that evolution of climatic niches and ecological divergence in the Amazonia‐open diagonal ecotone must have had an important role in the speciation of species of the group.

Analyzing whether species' niches evolve through conservatism or divergence is paramount to infer whether ecological speciation events occurred or not (Rato et al., 2015). The largest overlap found in species comparisons was between *K. altamazonica* and *K. pelviceps* (*D* = 0.54; although not significant), two species of the *calcarata* group mainly distributed west of the Amazon Basin (Figure 5), what may be suggestive of a niche conservatism involved in the speciation of closely related species. In most comparisons where *D* was close to 0 (i.e., low niche overlaps), we observed a strong tendency toward divergence between multidimensional spaces. However, some of the comparisons were not significant. Theodoridis, Randin, Broennimann, Patsiou, and Conti (2013) explain that observed very low overlap values within the multivariate space consistently fall within the 95% confidence limits of null distributions. Thus, the result of a niche divergence may reflect differences in spatial autocorrelation of climate variables between regions. This suggests that the ecological differences found between species are no more or less similar than expected due to climate differentiation but imply that these differences reflect the environmental heterogeneity between available habitats. Therefore, colonization of new environmental conditions has not been fully established, and other spatial factors (e.g., secondary dispersal) may also have played an important role in species divergence. Thus, spatial dispersal to occupy new habitats (as recovered in the BioGeoBEARS reconstruction) was also a major factor in species diversification and speciation. Sample size, background delimitation, and habitat availability can hinder powerful statistical inferences of niche evolution (Warren et al., 2008). Furthermore, niches analyzed here were measured in macroenvironmental scales, so there may still be niche differentiation occurring in micro‐environmental scales between the groups.

Significant niche divergence in the multidimensional space was observed for most of the comparisons between species from the nonsister groups *paulensis* and *striata* (e.g., *K. paulensis*—*K. striata*) and *paulensis* and *calcarata* (e.g., *K. altamazonica*—*K. vanzoi*), highlighting the *paulensis* group as a case of ecological (and possibly morphological) disparity in the evolution of the genus, as already observed for life‐history reproductive traits (Werneck et al., 2009). Additional studies are necessary to test morphological predictions, as species of this group have remarkable smaller body sizes than species from the other groups (Werneck et al., 2009). Thus, we argue that, after an initial divergence event, niche shifts and ecological divergence to the regional conditions of the Cerrado savannas were decisive to promote speciation of the *paulensis* species group. The expansion and establishment of Cerrado (as in other savannas of the world) occurred mostly during the late Miocene and Pliocene (Edwards et al., 2010), when vegetational and climatic differences were accentuated and favored niche divergence and speciation in *Kentropyx*. This period, congruent with the main diversification events of the *paulensis* group, was determinant for the establishment of the discordant regional conditions important for ecological divergence.

Pontes‐da‐Silva et al. (2018) showed that *K. calcarata* exhibits considerable geographic variation in thermal ecology and tolerance along the Amazonia‐Cerrado gradient, indicating intraspecific differences in climatic vulnerability that can result in diverse responses to historical and future climate change below the species level. Together with the niche dynamics presented here, this could result in evolutionary distinctiveness of speciation and adaptation processes and complementary conservation value and vulnerability of central and peripheral populations within *Kentropyx* spp. The fact that *Kentropyx* lizards are active thermoregulators (irrespective of the main vegetation coverage), somehow buffering environmental temperature variation, implies that species did not have historically to face substantial changes in behavioral thermal physiology when shifting between habitats ecologically so different as rainforest and savannas, for example. So, together with results from historical biogeography analyses that support dispersal scenarios, niche evolution results argue for a stronger signal for ecological speciation acting across spatiotemporal scales, rather than vicariant speciation, to generate the main pattern of recurrent habitat shifts during the genus diversification (Antonelli, Zizka, et al., 2018; Rheindt, Christidis, & Norman, 2008).

## 5 MAIN CONCLUSIONS

Our results inferred from large ecological and phylogenetic datasets indicate that a combination of historical geomorphological dispersal events and niche evolution (mostly ecological divergence for the *paulensis* species group) during the Miocene–Pliocene had pivotal roles in the speciation processes of this dynamic and conspicuous group of Neotropical lizards. The low levels of niche conservatism found likely facilitated dispersal along changing environments, promoting ecological divergent speciation and successful occupancy of new habitats. Species from the *paulensis* group have ecological associations markedly different from the other groups, representing a case of ecological disparity within the genus evolution, likely associated with adaptation to distinct regional ecological conditions during the Cerrado savanna establishment at the Late Miocene–Pliocene. These results suggest that *Kentropyx* spp. show signals of local adaptation to selective environmental pressures, a hypothesis passible to be tested with independent evidence.

Despite being the most comprehensive phylogenetic study for the genus, some questions remain open. We point to directions for future studies to increase understanding the evolutionary forces that drove diversification in *Kentropyx* and how future environmental change might impact diversity and potential adaptive responses within the genus. For example, further investigations of the genetic basis of phenotypic and ecological variation are needed to uncover the adaptive significance of niche evolution and if different species and populations can be impacted differently by climate change. Phylogenomics and demographic studies can help understanding the introgression signs in the *calcarata* species group; the evolution of parthenogenesis (*K. borckiana*); colonization history and population dynamics of *K. calcarata* populations in the Atlantic forest, *K. calcarata* in the Amazon/Cerrado borders; and *K. striata* in the Amazonian savannas. In summary, our study is consistent with the notion that ecological divergence and dispersal events associated with shifts between forests and savannas, rather than geographic isolation, can be more common as a mode of speciation in Neotropical lizards than previously thought.

## CONFLICT OF INTEREST

None declared.

## AUTHORS CONTRIBUTIONS

FPW and YS designed the research; YS generated the sequence data; MARJ provided geographical data; YS and JPZ analyzed data; TCAP, GRC, FPW, and MTR contributed samples; FPW contributed reagents, analysis, and publication tools; FPW and YS wrote the study, and all authors helped improving the final version.

## Data Availability

All sequence data were deposited in the GenBank, and supplementary tables and figures were deposited in the Dryad Digital Repository (https://doi.org/10.5061/dryad.zkh18936n).
