# Supplementary material for: The combined role of dispersal and niche evolution in the diversification of Neotropical lizards
Source: Ecol Evol. 2020 Feb 14;10(5):2608–25. doi: 10.1002/ece3.6091 (PMC7069304; doi:10.1002/ece3.6091)

SUPPORTING INFORMATION

The combined role of dispersal and niche evolution in the diversification of Neotropical lizards

SUPPORTING FIGURES

Fig S1. Nuclear gene tree generated by Bayesian inference from concatenated nuclear marker sequences SNCAIP, DNH3, RP40 and R35. Green and black balls indicate posterior probability above 75% and 90%, respectively.

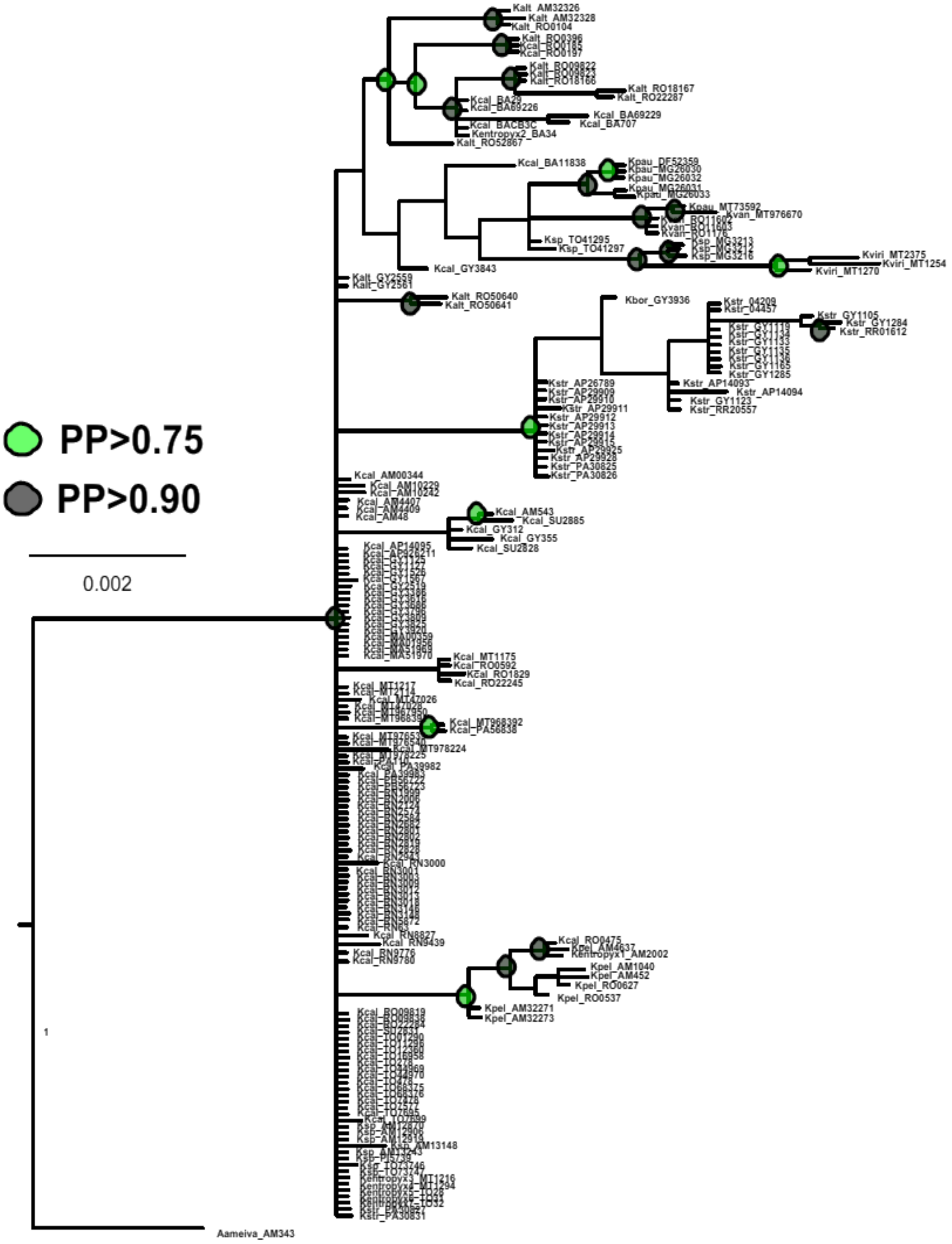

Supplement: Supplementary file 1 [file ECE3-10-2608-s001.pdf]
