# Supplementary material for: The combined role of dispersal and niche evolution in the diversification of Neotropical lizards
Source: Ecol Evol. 2020 Feb 14;10(5):2608–25. doi: 10.1002/ece3.6091 (PMC7069304; doi:10.1002/ece3.6091)
Supplement: Supplementary file 2 [file ECE3-10-2608-s002.pdf]

SUPPORTING INFORMATION

The combined role of dispersal and niche evolution in the diversification of Neotropical lizards

SUPPORTING FIGURES

Fig S2. Gene trees generated by Bayesian inference using the markers (a) SNCAIP, (b) DNH3; (c) RP40 and (d) R35. Green and black circles indicate posterior probability above 75% and 90%, respectively.

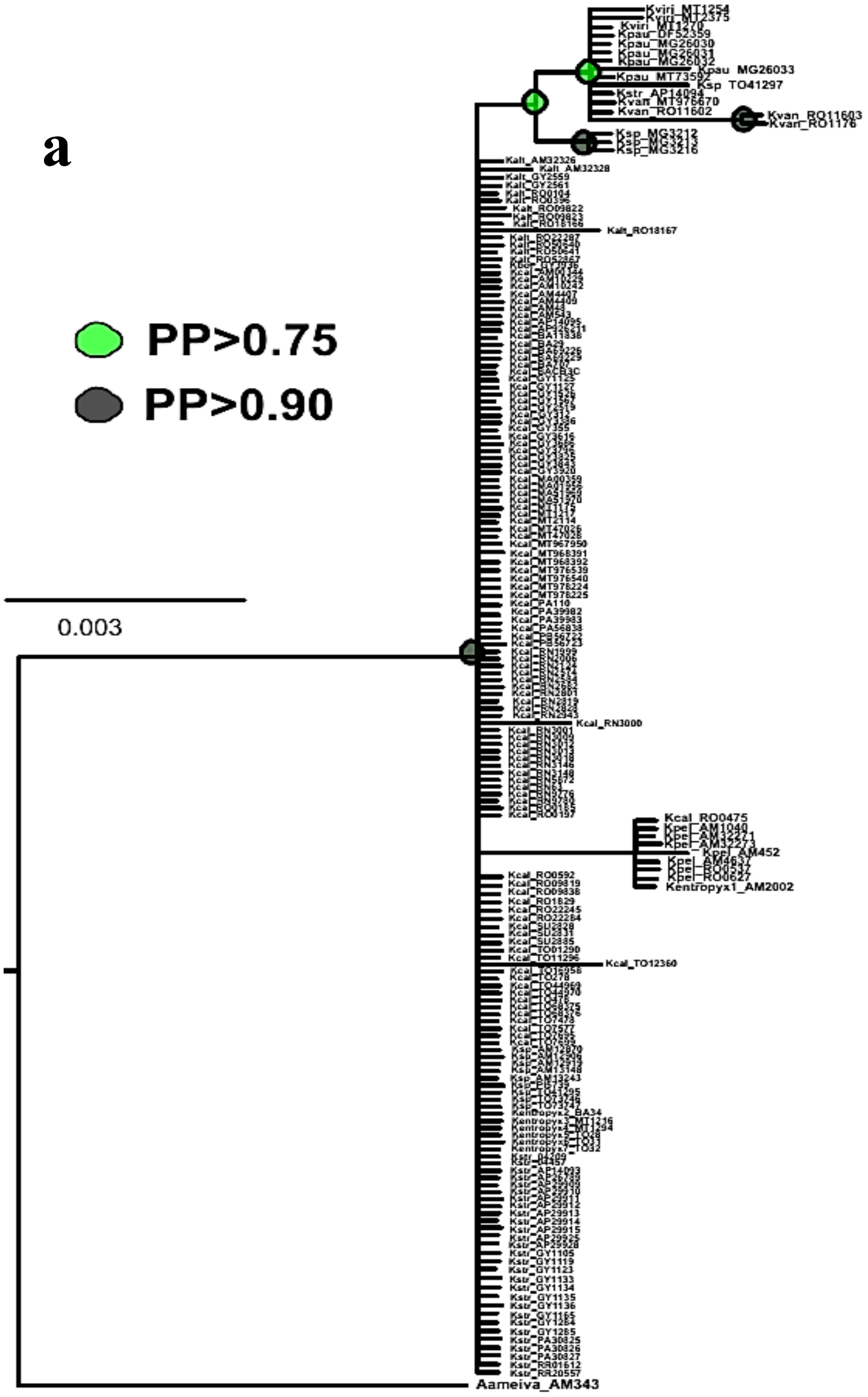

8

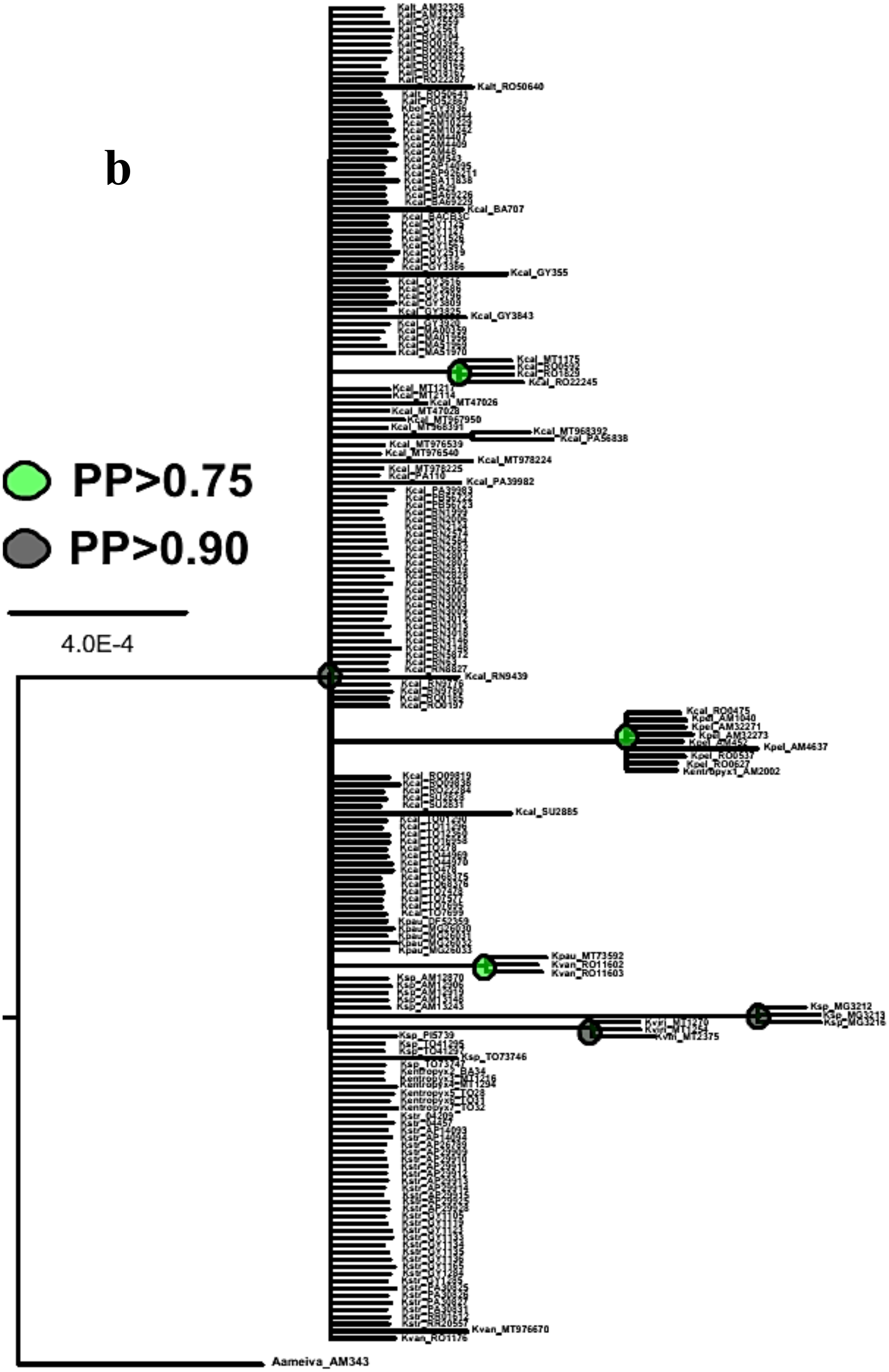
b
PP>0.75
PP>0.90
4.0E-4
Kalt_AM32326
Kalt_RO50640
Kcal_BA707
Kcal_GY355
Kcal_GY3843
Kcal_MT1175
Kcal_RO22245
Kcal_MT47026
Kcal_MT968392
Kcal_PA56838
Kcal_MT978224
Kcal_PA39982
Kcal_RN9439
Kcal_RO0475
Kpel_AM4637
Kcal_SU2885
Kpau_MT73592
Kvan_RO11602
Kvan_RO11603
Ksp_MG3212
Ksp_TO73746
Kvan_MT976670
Aameiva_AM343

0

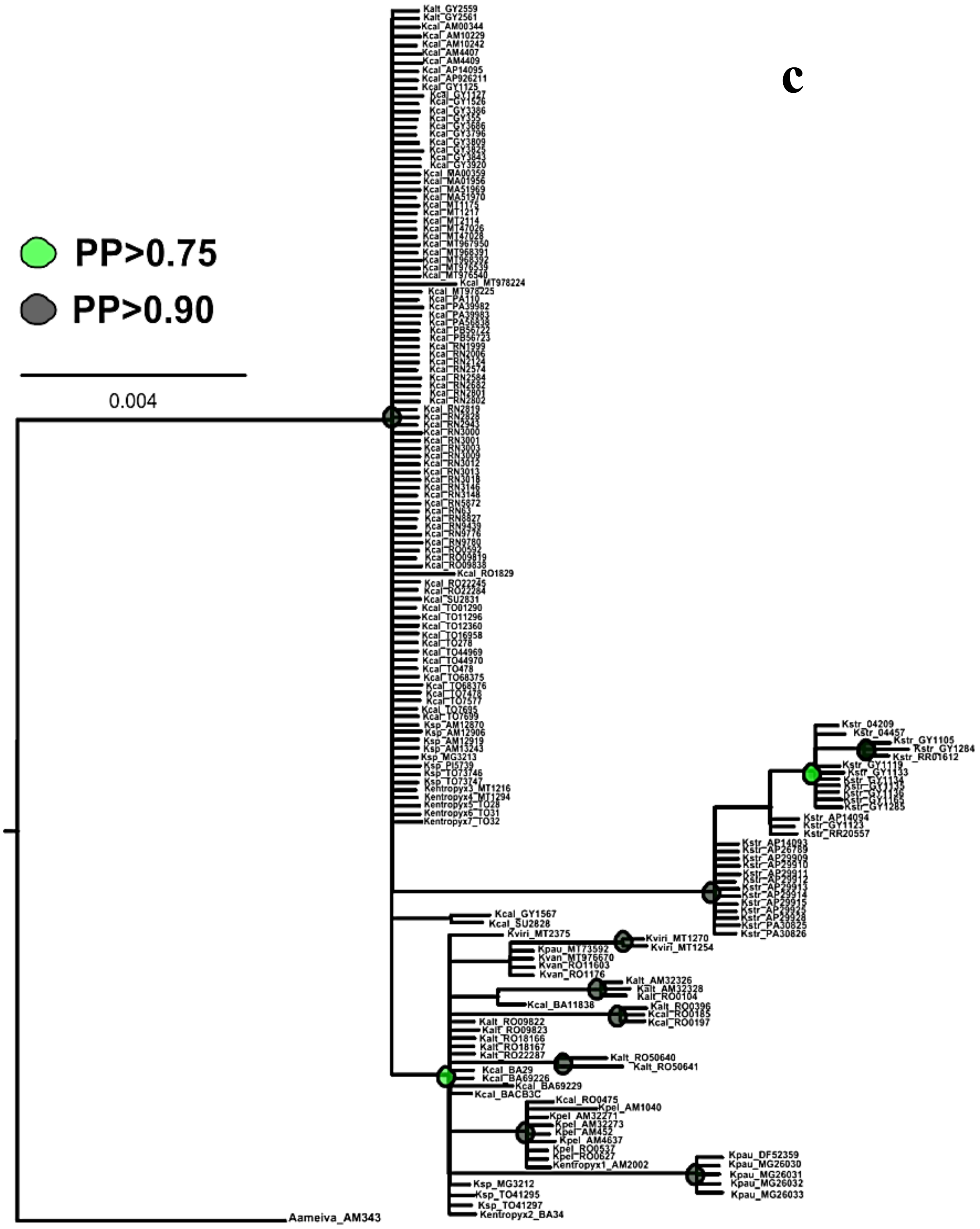

9

10

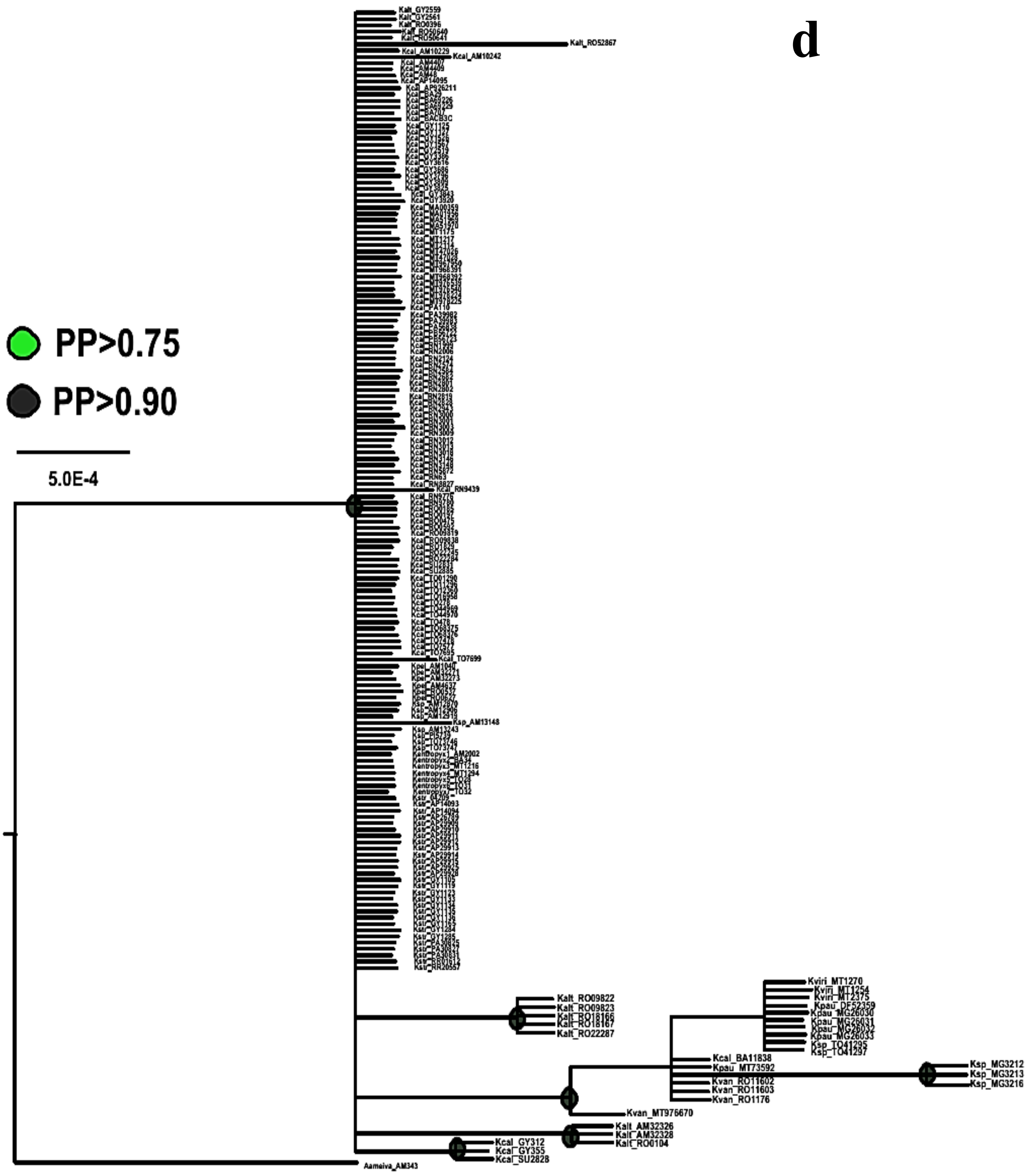
d
PP>0.75
PP>0.90
5.0E-4
Kalt_RO52867
Kcal_AM10242
Kcal_RN9439
Kcal_TO7699
Ksp_AM13148
Kalt_RO09822
Kalt_RO09823
Kalt_RO18166
Kalt_RO18167
Kalt_RO22287
Kviri_MT1270
Kviri_MT1254
Kviri_MT2375
Kpau_DF52359
Kpau_MG26030
Kpau_MG26031
Kpau_MG26032
Kpau_MG26033
Ksp_TO41295
Ksp_TO41297
Kcal_BA11838
Kpau_MT73592
Kvan_RO11602
Kvan_RO11603
Kvan_RO1176
Ksp_MG3212
Ksp_MG3213
Ksp_MG3216
Kvan_MT976670
Kalt_AM32326
Kalt_AM32328
Kalt_RO0104
Kcal_GY312
Kcal_GY355
Kcal_SU2828
Aameiva_AM343
