# Supplementary material for: The combined role of dispersal and niche evolution in the diversification of Neotropical lizards
Source: Ecol Evol. 2020 Feb 14;10(5):2608–25. doi: 10.1002/ece3.6091 (PMC7069304; doi:10.1002/ece3.6091)

SUPPORTING INFORMATION

The combined role of dispersal and niche evolution in the diversification of Neotropical lizards

SUPPORTING FIGURES

Fig S3. Species tree and biogeographic reconstruction with all possible ancestral areas inferred under the DEC + J model presented as pie charts on each node.

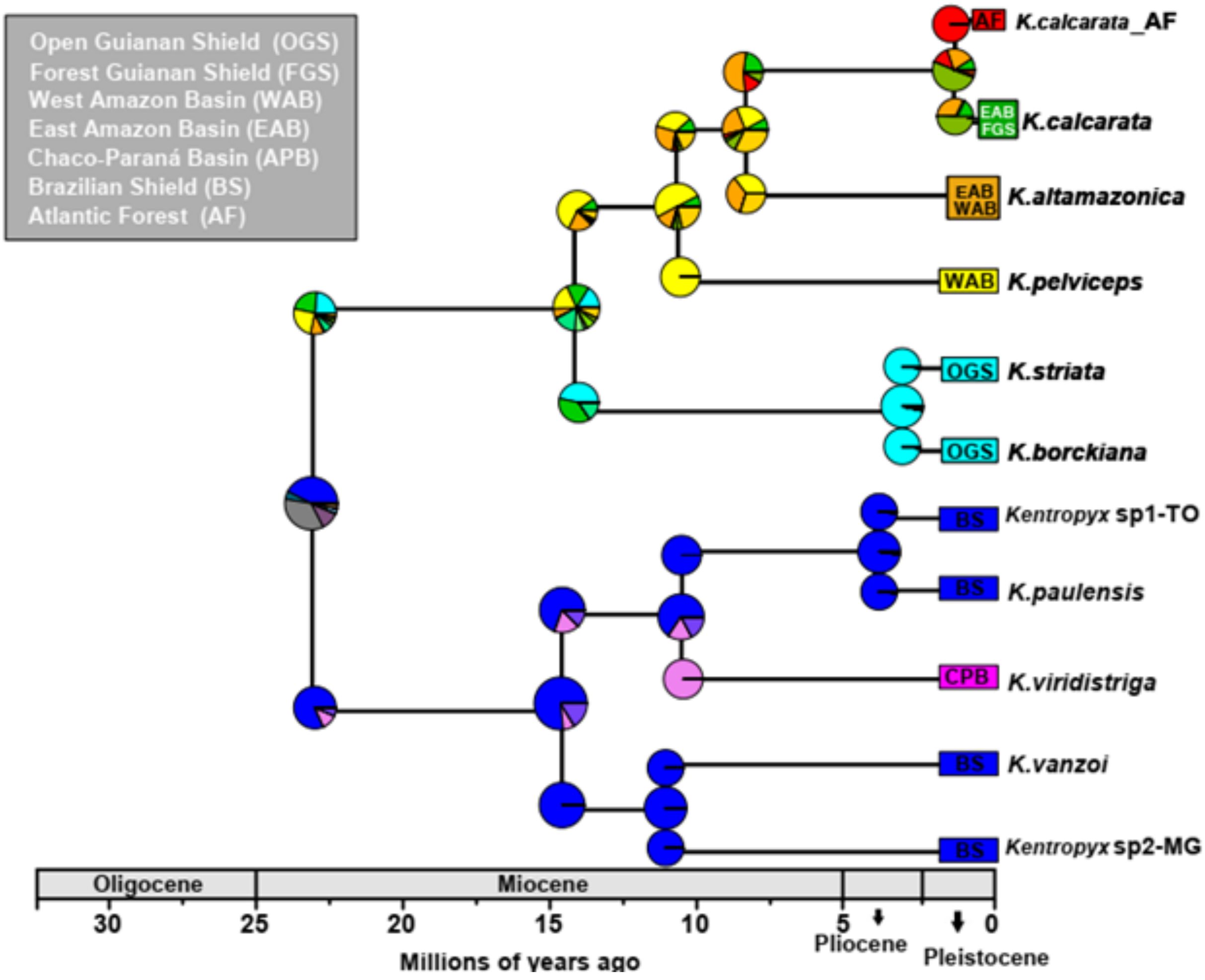

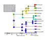

Supplement: Supplementary file 3 [file ECE3-10-2608-s003.pdf]
