# Supplementary material for: The combined role of dispersal and niche evolution in the diversification of Neotropical lizards
Source: Ecol Evol. 2020 Feb 14;10(5):2608–25. doi: 10.1002/ece3.6091 (PMC7069304; doi:10.1002/ece3.6091)
Supplement: Supplementary file 4 [file ECE3-10-2608-s004.pdf]

SUPPORTING INFORMATION

The combined role of dispersal and niche evolution in the diversification of Neotropical lizards

SUPPORTING FIGURES

Fig S4. Predicted niche occupancy profiles (PNO) for the nine species of *Kentropyx*. PNOs of the 12 variables used in the construction of niche models in which the horizontal axis of the PNO represents the occupation of each variable and the vertical axis represents the suitability of each species for each variable presented. Abbreviation: Kalt: *K. altamazonica*; Kbor: *K. borckiana*; Kcal: *K. calcarata*; Kcal_AF: Atlantic Forest populations of *K. calcarata*; Kpau: *K. paulensis*; Kpel: *K. pelviceps*; Ksp: *K.* sp; Kstr: *K. striata*; Kvan: *K. vanzoi* and Kviri: *K. viridistriga*

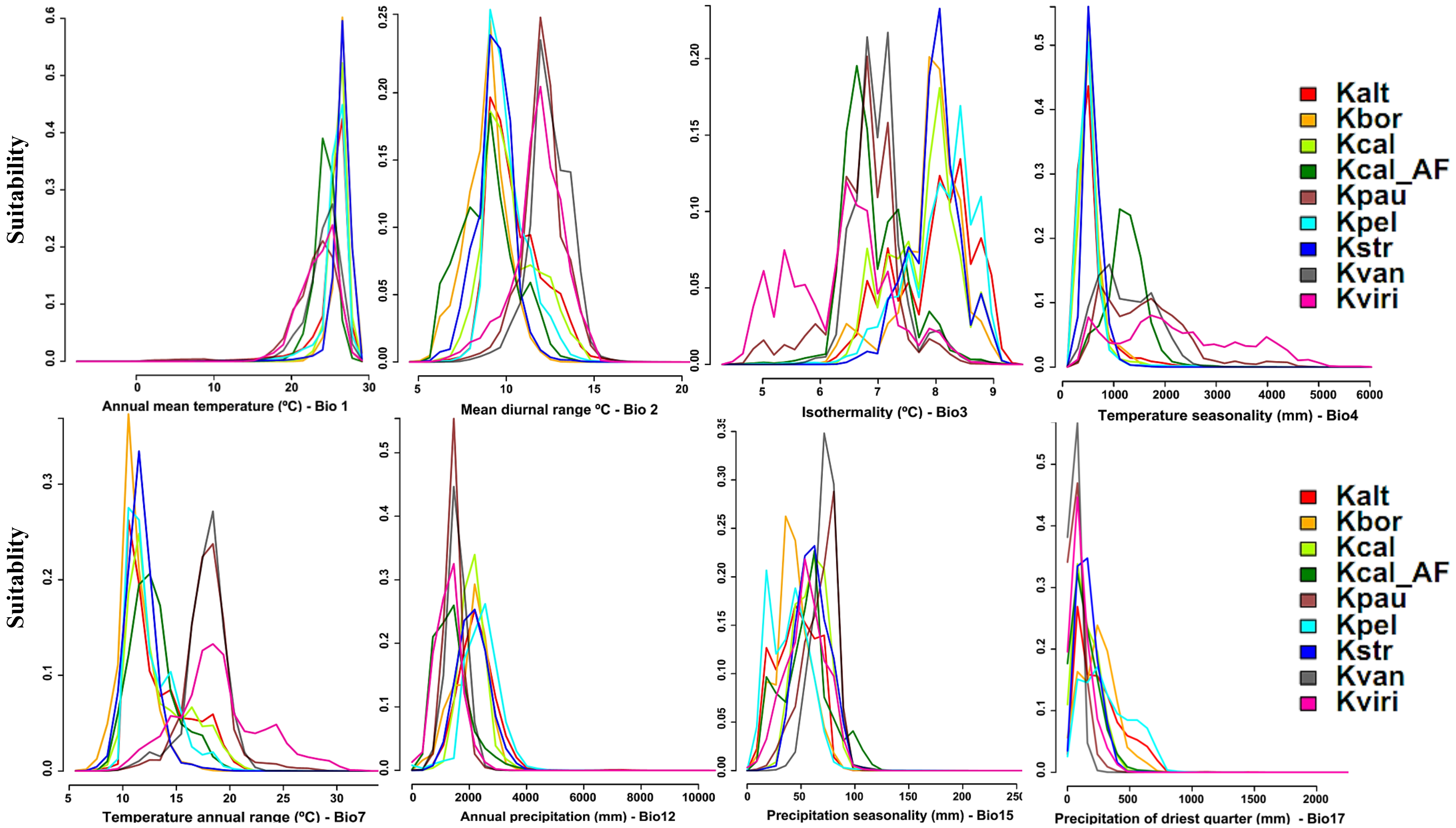

Suitability
Annual mean temperature (ºC) - Bio 1
Mean diurnal range ºC - Bio 2
Isothermality (ºC) - Bio3
Temperature seasonality (mm) - Bio4
Temperature annual range (ºC) - Bio7
Annual precipitation (mm) - Bio12
Precipitation seasonality (mm) - Bio15
Precipitation of driest quarter (mm) - Bio17
Kalt
Kbor
Kcal
Kcal_AF
Kpau
Kpel
Kstr
Kvan
Kviri

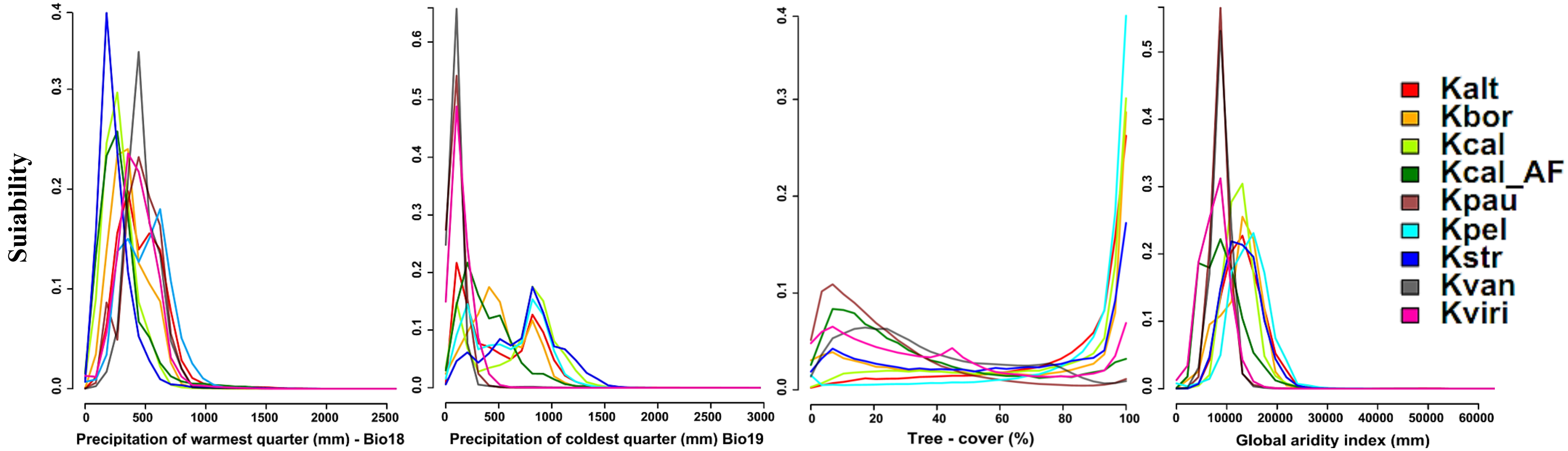

Suiability
Precipitation of warmest quarter (mm) - Bio18
Precipitation of coldest quarter (mm) Bio19
Tree - cover (%)
Global aridity index (mm)
Kalt
Kbor
Kcal
Kcal_AF
Kpau
Kpel
Kstr
Kvan
Kviri
