# Supplementary material for: The combined role of dispersal and niche evolution in the diversification of Neotropical lizards
Source: Ecol Evol. 2020 Feb 14;10(5):2608–25. doi: 10.1002/ece3.6091 (PMC7069304; doi:10.1002/ece3.6091)
Supplement: Supplementary file 5 [file ECE3-10-2608-s005.pdf]

SUPPORTING INFORMATION

The combined role of dispersal and niche evolution in the diversification of Neotropical lizards

SUPPORTING FIGURES

Fig S5. Ancestral reconstruction of the 12 variables used to construct the niche models for *Kentropyx*. We integrate the proposed phylogenetic hypothesis based on the species tree and the PNOs for the reconstruction of average maximum tolerance climatic based on 1000 random repetitions of the PNOs on the internal nodes. The point on the dashed vertical line corresponds to the average of 80% of the central density for each species. Abbreviation: Kalt: *K. altamazonica*; Kbor: *K. borckiana*; Kcal: *K. calcarata*; Kcal_AF: Atlantic Forest *K. calcarata*_AF; Kpau: *K. paulensis*; Kpel: *K. pelviceps*; Ksp: *K.* sp; Kstr: *K. striata*; Kvan: *K. vanzoi* and Kviri: *K. viridistriga.*

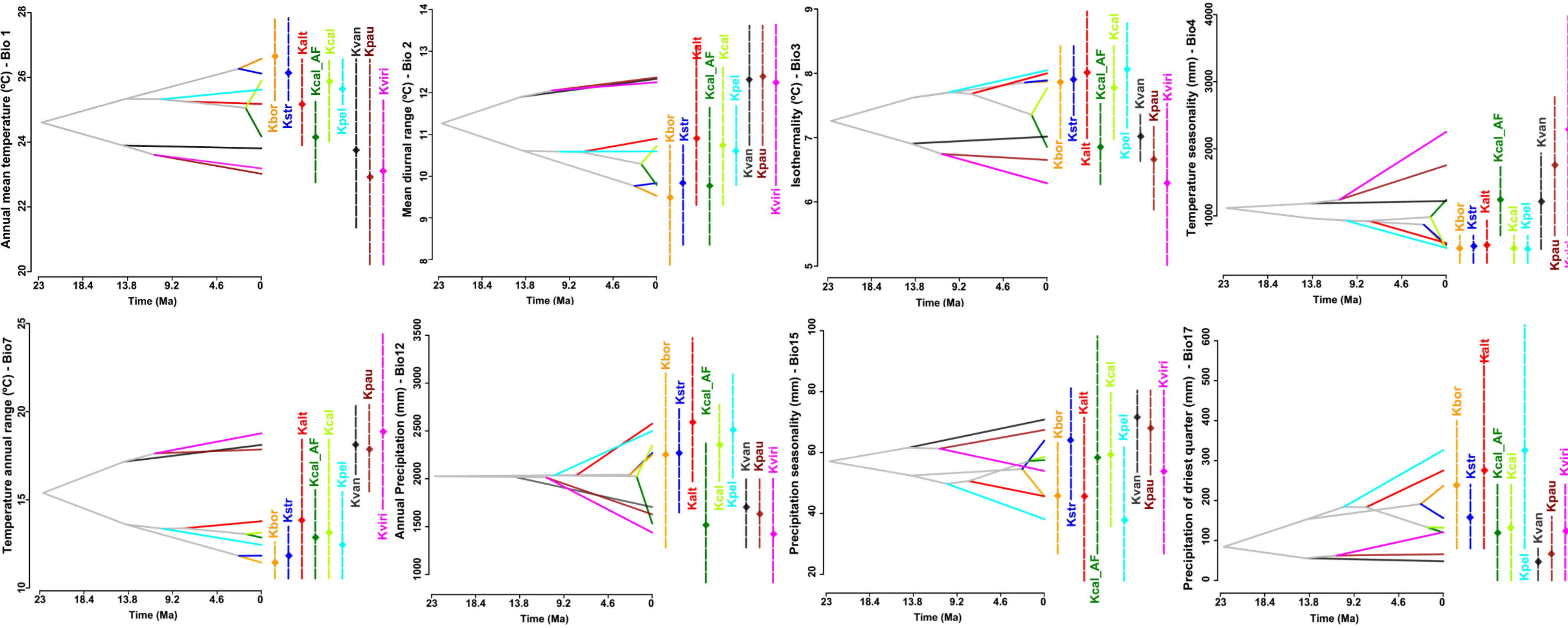

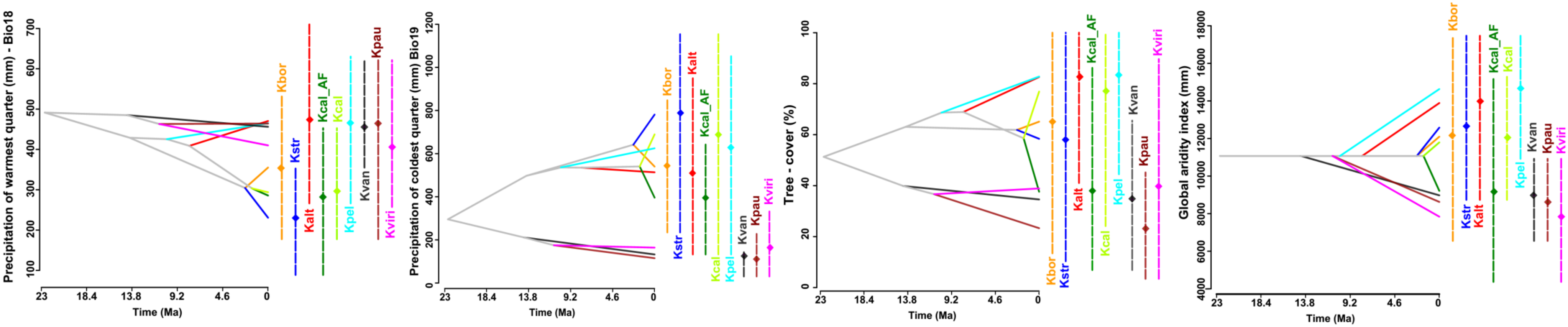
Precipitation of warmest quarter (mm) - Bio18
Time (Ma)
Precipitation of coldest quarter (mm) Bio19
Time (Ma)
Tree - cover (%)
Time (Ma)
Global aridity index (mm)
Time (Ma)
Kbor
Kstr
Kalt
Kcal_AF
Kcal
Kpel
Kvan
Kpau
Kviri
