# Supplementary material for: The combined role of dispersal and niche evolution in the diversification of Neotropical lizards
Source: Ecol Evol. 2020 Feb 14;10(5):2608–25. doi: 10.1002/ece3.6091 (PMC7069304; doi:10.1002/ece3.6091)
Supplement: Supplementary file 6 [file ECE3-10-2608-s006.pdf]

SUPPORTING INFORMATION

The combined role of dispersal and niche evolution in the diversification of Neotropical lizards

SUPPORTING TABLES

Table S1. Details of the samples used for molecular data collecting, with locality data for sequenced samples, identification number, longitude and latitude. * Outgroup; Abbreviation of states: Amapá (AP), Amazonas (AM), Bahia (BA), Maranhão (MA), Mato Grosso (MT), Mato Grosso do Sul (MS), Minas Gerais (MG), Pará (PA), Paraíba (PB), Piauí (PI), Rio Grande do Norte (RN), Rondônia (RO), Roraima (RR) and Tocantins (TO).

Taxon	Locality	ID Number	Longitude	Latitude
*A. ameiva**	Reserva da Campina, AM	FPWerneck00343	-59.7002794	-2.6986264
K. altamazonica	Alta Floresta D'Oeste, RO	CHUNB52867	-60.277528	-12.121981
K. altamazonica	Cerejeiras, RO	CHUNB50640	-60.8122	-13.1889
K. altamazonica	Cerejeiras, RO	CHUNB50641	-60.8122	-13.1889
K. altamazonica	Guajará-Mirim, RO	CHUNB22287	-60.938331	-11.862561
K. altamazonica	Humaitá, AM	CHUNB32326	-64.041244	-8.814814
K. altamazonica	Humaitá, AM	CHUNB32328	-64.041244	-8.814814
K. altamazonica	Pimenta Bueno, RO	CHUNB18166	-62.196175	-12.011539
K. altamazonica	Pimenta Bueno, RO	CHUNB18167	-62.196175	-12.011539
K. altamazonica	Vilhena, RO	CHUNB09822	-60.275	-12.0205555
K. altamazonica	Vilhena, RO	CHUNB09823	-60.275	-12.0205555
K. altamazonica	Porto Velho, RO	HJ0104	-64.900006	-11.400117
K. altamazonica	Porto Velho, RO	HJ0396	-64.900006	-11.400117
K. altamazonica	Kuribrang Camp, Guyana	BPN2559	-59.533194	5.396639
K. altamazonica	Kuribrang Camp, Guyana	BPN2561	-59.533194	5.396639
K. borckiana	Demerara-Mahaica, Guyana	PK3936	-58.09820	6.5464260
K. calcarata	Alta Floresta, MT	CHUNB47026	-55.917445	-6.2222
K. calcarata	Alta Floresta, MT	CHUNB47028	-55.917445	-6.800000

K. calcarata	Amapá, AP	CHUNB14095	-50.790564	-6.800000
K. calcarata	Carolina, MA	CHUNB51969	-47.261630	-12.770000
K. calcarata	Carolina, MA	CHUNB51970	-47.261630	-12.770000
K. calcarata	Caseara, TO	CHUNB44969	-49.883917	-9.36986388
K. calcarata	Caseara, TO	CHUNB44970	-49.883917	-9.36986388
K. calcarata	Guajará-Mirim, RO	CHUNB22245	-64.900006	-10.309439
K. calcarata	Guajará-Mirim, RO	CHUNB22284	-64.900006	-10.309439
K. calcarata	Jacareacanga, PA	CHUNB56838	-57.7528	-10.309439
K. calcarata	Novo Progresso, PA	CHUNB39982	-54.9133138	-15.188121
K. calcarata	Novo Progresso, PA	CHUNB39983	-54.9133138	-8.833333
K. calcarata	Palmas, TO	CHUNB11296	-48.345922	-8.833333
K. calcarata	Palmas, TO	CHUNB12360	-48.345922	-9.750000
K. calcarata	Palmas, TO	CHUNB16958	-48.345922	-9.750000
K. calcarata	Ribeirão Cascalheira, MT	CHUNB68375	-51.820000	-14.800000
K. calcarata	Ribeirão Cascalheira, MT	CHUNB68376	-51.820000	-15.116667
K. calcarata	Vilhena, RO	CHUNB09819	-60.277528	-6.465278
K. calcarata	Vilhena, RO	CHUNB09838	-60.277528	-6.3258333
K. calcarata	Guaraí, TO	MTR7478	-48.516667	-7.229730
K. calcarata	Guaraí, TO	MTR7577	-48.516667	-7.771389
K. calcarata	UHE Lajeado, TO	LAJ278	-48.350000	-6.589167
K. calcarata	UHE Lajeado, TO	LAJ478	-48.350000	-10.300000
K. calcarata	UHE Guaporé, MT	RGL1217	-58.966667	-7.216667
K. calcarata	Canaã dos Carajás, PA	110	-50.077222	-7.216667
K. calcarata	Cachoeira das Pombas, AM	MTR10242	-60.3463889	-9.600000
K. calcarata	Cachoeira das Pombas, AM	MTR10229	-60.3463889	-9.600000
K. calcarata	Carolina, MA	ESTR00359	-47.261630	-9.538739
K. calcarata	Palmeiras do Tocantins, TO	ESTR01290	-48.317778	-9.633333
K. calcarata	Estreito, MA	ESTR01956	-47.460000	0.983333
K. calcarata	Montenegro, RO	UNIBAN1829	-63.200000	-10.250833
K. calcarata	Babaçulândia, TO	MTR7695	-47.766667	-9.566667

K. calcarata	Babaçulândia, TO	MTR7699	-47.766667	-9.566667
K. calcarata	Porto Velho, RO	HJ0185	-64.500000	-13.350000
K. calcarata	Porto Velho, RO	HJ0197	-64.500000	-13.350000
K. calcarata	Abunã, RO	HJ0475	-65.334350	-14.800000
K. calcarata	Mutum, RO	HJ0592	-64.950000	-10.016667
K. calcarata	Serra do Navio, AP	926211	-50.050000	-10.016667
K. calcarata	Aripuanã, MT	967950	-59.549167	-15.116667
K. calcarata	Aripuanã, MT	LG1175	-59.549167	3.206167
K. calcarata	Apiacás, MT	968391	-57.383333	3.206167
K. calcarata	Apiacás, MT	968392	-57.383333	-17.108700
K. calcarata	Gaúcha do Norte, MT	976540	-53.433333	-17.108700
K. calcarata	Gaúcha do Norte, MT	976539	-53.433333	-17.108700
K. calcarata	Vila Rica, MT	978224	-51.233333	-9.578084
K. calcarata	Vila Rica, MT	978225	-51.233333	-9.578084
K. calcarata	UHE Guaporé, MT	LG2114	-58.966667	2.035456
K. calcarata	Takutu-Upper Essequibo, Guyana	AMS312	-59.405533	-7.229730
K. calcarata	Takutu-Upper Essequibo, Guyana	AMS355	-59.405533	-7.229730
K. calcarata	Patawa, French Guyana	BPN1526	-52.452030	4.548170
K. calcarata	Patawa, French Guyana	BPN1567	-52.452030	4.548170
K. calcarata	Suriname	BPN2519	-59.533520	5.396670
K. calcarata	Suriname	BPN2828	-55.629410	2.477000
K. calcarata	Suriname	BPN2831	-55.629410	2.477000
K. calcarata	Suriname	BPN2885	-55.630210	2.466490
K. calcarata	Potaro-Siparuni, Guyana	BPN3386	-58.921602	4.413793
K. calcarata	Potaro-Siparuni, Guyana	BPN3616	-58.921602	4.413793
K. calcarata	Potaro-Siparuni, Guyana	BPN3796	-59.643278	5.001869
K. calcarata	Potaro-Siparuni, Guyana	BPN3825	-59.643278	5.001869
K. calcarata	Potaro-Siparuni, Guyana	TJC1125	-59.516070	5.275050
K. calcarata	Potaro-Siparuni, Guyana	TJC1127	-59.516070	5.275050
K. calcarata	Potaro-Siparuni, Guyana	PK3686	-58.728611	-4.731944

K. calcarata	Potaro-Siparuni, Guyana	PK3809	-58.783333	-4.412778
K. calcarata	Potaro-Siparuni, Guyana	PK3843	-58.712778	-4.732778
K. calcarata	Potaro-Siparuni, Guyana	PK3920	-59.026111	-4.746944
K. calcarata	São José do Jabote, AM	HT4407	-65.21484	-2.163792
K. calcarata	São João do Lago da Velha, AM	HT4409	-65.21484	-2.163792
K. calcarata	Caracaraí, RR	Viruá 543	-61.13463	1.815873
K. calcarata	São José do Jabote, Urucará, AM	CTGA048	-58.20637	-2.442202
K. calcarata	Reserva da Campina, AM	FPW00344	-59.7002794	-2.698626
K. calcarata	Tailândia, PA	1999	-51.20728	-0.516350
K. calcarata	Tailândia, PA	2006	-51.20728	-0.516350
K. calcarata	Tailândia, PA	2124	-51.20728	-0.516350
K. calcarata	São Sebastião, AM	MTR12870	-58.95898	-1.916757
K. calcarata	Igarapé Tarumã-Açu, AM	MTR12906	-58.95898	-2.980756
K. calcarata	São Sebastião, AM	MTR12919	-58.95898	-1.916757
K. calcarata	Pacamiri, AM	MTR13148	-58.22055	-4.596944
K. calcarata	Palhalzinho, AM	MTR13243	-57.46547	-4.481279
*K. calcarata*_AF	Mamanguape, PB	CHUNB56723	-35.200000	-12.940000
*K. calcarata*_AF	Mamanguape, PB	CHUNB56722	-35.200000	-12.940000
*K. calcarata*_AF	Maragogipe, BA	CHUNB69226	-38.910000	-12.121981
*K. calcarata*_AF	Maragogipe, BA	CHUNB69229	-38.910000	-12.121981
*K. calcarata*_AF	Una, BA	MD2707	-39.197266	-6.3258333
*K. calcarata*_AF	Ilhéus, BA	ABA29	-39.050000	-14.300000
*K. calcarata*_AF	Itacaré, BA	MTR11838	-39.000000	-10.250833
*K. calcarata*_AF	Ilhéus, BA	CB3C	-39.050000	-17.108700
*K. calcarata*_AF	Nísia Floresta, RN	8827	-35.05618	-6.253352
*K. calcarata*_AF	Canguaretama, RN	2574	-35.04431	-6.296736
*K. calcarata*_AF	Canguaretama, RN	2584	-35.04431	-6.296736
*K. calcarata*_AF	Canguaretama, RN	2682	-35.04431	-6.296736
*K. calcarata*_AF	Goianinha, RN	2801	-35.06531	-6.253453
*K. calcarata*_AF	Goianinha, RN	2802	-35.06531	-6.253453

*K. calcarata*_AF	Goianinha, RN	2819	-35.06531	-6.253453
*K. calcarata*_AF	Espírito Santo, RN	2828	-35.22139	-6.196379
*K. calcarata*_AF	Arês, RN	2943	-35.22139	-6.196379
*K. calcarata*_AF	Canguaretama, RN	3001	-35.04431	-6.296736
*K. calcarata*_AF	Goianinha, RN	3012	-35.06531	-6.253453
*K. calcarata*_AF	Arês, RN	3000	-35.22139	-6.196379
*K. calcarata*_AF	Canguaretama, RN	3003	-35.04431	-6.296736
*K. calcarata*_AF	Goianinha, RN	3013	-35.06531	-6.253453
*K. calcarata*_AF	Goianinha, RN	3018	-35.04431	-6.296736
*K. calcarata*_AF	Goianinha, RN	3146	-35.06531	-6.253453
*K. calcarata*_AF	Goianinha, RN	3148	-35.06531	-6.253453
*K. calcarata*_AF	Aripuanã, RN	63	-35.05027	-6.378376
*K. calcarata*_AF	Baía Formosa, RN	9776	-35.07193	-6.361787
*K. calcarata*_AF	Baía Formosa, RN	9780	-35.07193	-6.361787
*K. calcarata*_AF	Nísia Floresta, RN	9439	-35.05618	-6.253352
*K. calcarata*_AF	Baía Formosa, RN	3009	-35.05618	-6.253352
*K. calcarata*_AF	Baía Formosa, RN	5872	-35.05618	-6.253352
*K. calcarata*_AF	Uruçuí-Una, Uruçuí, PI	MTR5739	-45.25903	-7.329778
K. paulensis	Brasília, DF	CHUNB52359	-47.921386	-7.229730
K. paulensis	Ribeirão Cascalheira, MT	CHUNB73592	-51.82	-7.229730
K. paulensis	Paracatu, MG	CHUNB26030	-46.871860	-9.631667
K. paulensis	Paracatu, MG	CHUNB26031	-46.871860	-9.631667
K. paulensis	Paracatu, MG	CHUNB26032	-46.871860	-11.400117
K. paulensis	Paracatu, MG	CHUNB26033	-46.871860	-11.400117
K. pelviceps	Humaitá, AM	CHUNB32273	-63.11677	-7.572392
K. pelviceps	Humaitá, AM	CHUNB32271	-63.11677	-7.572392
K. pelviceps	Mutum, RO	HJ0537	-63.70227	-8.533491
K. pelviceps	Caiçara, RO	HJ0627	-63.90043	-8.7611605
K. pelviceps	Roraima, RR	HT4637	-63.61083	-5.3972734
K. pelviceps	São João do Lago da Velha, AM	CTGA452	-65.11596	-7.514981

K. pelviceps	Rio Japurá, AM	CTGA1040	-67.25497	-2.011626
K. striata	Amapá, AP	CHUNB14094	-50.90000	0.300000
K. striata	Amapá, AP	CHUNB14093	-50.90000	0.300000
K. striata	Boa vista, RR	CHUNB01612	-60.80021	3.300111
K. striata	Monte Alegre, PA	CHUNB30825	-54.18823	-2.051911
K. striata	Monte Alegre, PA	CHUNB30831	-54.18823	-2.051911
K. striata	Monte Alegre, PA	CHUNB30827	-54.18823	-2.051911
K. striata	Monte Alegre, PA	CHUNB30826	-54.18823	-2.051911
K. striata	Imbaimadai, Guyana	BPN1105	-60.29691	5.703194
K. striata	Imbaimadai, Guyana	BPN1119	-60.29691	5.703194
K. striata	Imbaimadai, Guyana	BPN1123	-60.29691	5.703194
K. striata	Imbaimadai, Guyana	BPN1133	-60.29691	5.703194
K. striata	Imbaimadai, Guyana	BPN1134	-60.29691	5.703194
K. striata	Imbaimadai, Guyana	BPN1135	-60.29691	5.703194
K. striata	Imbaimadai, Guyana	BPN1136	-60.29691	5.703194
K. striata	Imbaimadai, Guyana	BPN1165	-60.29691	5.703194
K. striata	Imbaimadai, Guyana	BPN1284	-60.29691	5.703194
K. striata	Imbaimadai, Guyana	BPN1285	-60.29691	5.703194
K. striata	Almeirim, PA	MPEG26789	-52.40776	-1.384589
K. striata	Mazagão, PA	MPEG29909	-51.83021	-0.333333
K. striata	Mazagão, PA	MPEG29910	-51.83021	-0.333333
K. striata	Mazagão, PA	MPEG29911	-51.83021	-0.333333
K. striata	Mazagão, PA	MPEG29912	-51.83021	-0.333333
K. striata	Mazagão, PA	MPEG29913	-51.83021	-0.333333
K. striata	Mazagão, PA	MPEG29914	-51.83021	-0.333333
K. striata	Mazagão, PA	MPEG29915	-51.83021	-0.333333
K. striata	Mazagão, PA	MPEG29928	-51.83021	-0.333333
K. striata	Mazagão, PA	MPEG29925	-51.83021	-0.333333
K. striata	Ilha de Maracá, RR	MTR20557	-61.03333	1.7750000
K. vanzoi	Vilhena, RO	CHUNB11602	-60.145800	-12.740600

K. vanzoi	Vilhena, RO	CHUNB11603	-60.145800	-12.740600
K. vanzoi	Vilhena, RO	LG1176	-60.100000	-12.700000
K. vanzoi	Gaúcha do Norte, MT	976670	-53.433333	-13.350000
K. viridistriga	Fazenda Perseverança, Bolívia	UFMT1254	-	-
K. viridistriga	Corumbá, MS	UFMT1270	-56,7233	-18,731
K. viridistriga	Poconé, MT	UFMT2375	-56,9485	-16,7845
Kentropyx sp. 1	Mateiros, TO	CHUNB41295	-46.672089	-17.108700
Kentropyx sp. 1	Mateiros, TO	CHUNB41297	-46.672089	-17.108700
Kentropyx sp. 1	Pium, TO	CHUNB73746	-49.180397	-17.108700
Kentropyx sp. 1	Pium, TO	CHUNB73747	-49.180397	-17.108700
Kentropyx sp. 2	Diamantina, MG	RAB3212	-43.786869	-9.578084
Kentropyx sp. 2	Diamantina, MG	RAB3213	-43.786869	-9.578084
Kentropyx sp. 2	Diamantina, MG	RAB3216	-43.786869	2.035456
