# Supplementary material for: The combined role of dispersal and niche evolution in the diversification of Neotropical lizards
Source: Ecol Evol. 2020 Feb 14;10(5):2608–25. doi: 10.1002/ece3.6091 (PMC7069304; doi:10.1002/ece3.6091)
Supplement: Supplementary file 7 [file ECE3-10-2608-s007.pdf]

SUPPORTING INFORMATION

The combined role of dispersal and niche evolution in the diversification of Neotropical lizards

SUPPORTING TABLES

Table S2. Primers used for amplification and sequencing of the six loci used in this study. Each primer presented contains the source of origin and the annealing temperature (T) in degrees Celsius (° C).

Locus	Primers	Source	T
cyt *b*	IgCytb_F2 5’ CCA CCG TTG TTA TTC AAC TAC 3’<br>CB3-H 5’ GGC AAA TAG GAA RTA TCA TTC 3’	Palumbi et al., (1991)	46
16S	16SL 5’ CGC CTG TTT ATC AAA AAC AT 3’<br>16SH 5’ CCG GTC TGA ACT CAG ATC ACG T 3’	Palumbi et al., (1991)	54
SNCAIP	SNCAIP_F10 5’ CGC CAG YTG YTG GGR AAR GAW AT 3’<br>SNCAIP_R13 5’ GGW GAY TTG AGD GCA CTC TTR GGR CT 3’	Townsend et al., (2008)	57
DNH3	DNH3_f1 5’ GGT AAA ATG ATA GAA GAY TAC TG-3’<br>DNH3_r6 5’ CTK GAG TTR GAH ACA ATK ATG CCAT 3’	Townsend et al., (2008)	54
RP40	RP40f 5’ ATG TGG TGG ATG YTG GCT CGT 3’<br>RP40r 5’ GCT TCT CAG CWG CRG CCT GC 3’	Tod Reeder, from Oliveira et al., (2015)	57
R35	R35f 5’ CCA GTG AGC ATG ATA TGGG 3’<br>R35r 5’ GTT TCT GAC CAA AYT CCA CAGT 3’	Frank Burbrink, from Oliveira et al., (2015)	50

The final volume used for 15 μL, containing: 6,7 μL of ddH2O; 1,5 μL of MgCl2 (25mM); 1,5 μL dNTP (10 mM); 2 μL 10X Buffer (100mM Tris-HCl, 500mM KCl); 1 μL F primer (10 μM); 1 μL R primer (10 μM); 0,3 μL of Taq DNA Polimerase (5 U/μl) e 1μl de of the genomic DNA.

REFERENCES

Palumbi, S., Martin, A., Romano, S., McMillan, W., Stick, L. and Grabowski, G. (1991): The Single Tools Guide to PCR. Marine Version Honolulu, Hawaii

Oliveira, E. F., M. Gehara, V. a São-Pedro, X. Chen, E. a Myers, F. T. Burbrink, D. O. Mesquita, A. a Garda, G. R. Colli, M. T. Rodrigues, F. J. Arias, H. Zaher, R. M. L. Santos, and G. C. Costa. 2015. Speciation with gene flow in whiptail lizards from a Neotropical xeric biome. Mol. Ecol. 24:5957–75.

Townsend, T. M., Alegre, R. E., Kelley, S. T., Wiens, J. J. and Reeder, T. W. (2008). Rapid developmental of multiple nuclear loci for phylogenetic analysis using genomic resources: an example from squamate reptiles. Molecular Phylogenetics and Evolution, 47, 129–142.
