# Supplementary material for: The combined role of dispersal and niche evolution in the diversification of Neotropical lizards
Source: Ecol Evol. 2020 Feb 14;10(5):2608–25. doi: 10.1002/ece3.6091 (PMC7069304; doi:10.1002/ece3.6091)
Supplement: Supplementary file 10 [file ECE3-10-2608-s010.pdf]

SUPPORTING INFORMATION

The combined role of dispersal and niche evolution in the diversification of Neotropical lizards

SUPPORTING TABLES

Table S5. Occurrence points sample sizes and predictors (bioclimatic, vegetation, and aridity index) used to construct niche models for nine species of the lizard genus *Kentropyx*. The AUC values and the relative contribution of each predictor are an average for 20 replicates. Abbreviation of the species in Kalt: *K. altamazonica*; Kbor: *K. borckiana*; Kcal: *K. calcarata*; K.cal_AF: Atlantic Forest populations of *K. calcarata*; Kpau: *K. paulensis*; Kpel: *K. pelviceps*; Kstr: *K. striata*; Kvan: *K. vanzoi* and K.viri*: K. viridistriga*. . Parameters selected for each species and model performance are also depicted as following: Feature Classes-FC combinations (L = linear, Q = quadratic, H = hinge, P = product and T = threshold), Regularization Multipliers-RM. Model goodness-of-fit and performance as estimated by Akaike information criterion corrected for small sample sizes (AICc) and area under the curve of the receiver operating characteristic plot based on the testing data (AUC_{TEST}) and on the difference between training and testing AUC (AUC_{DIFF} - a overfitting quantification).

	**Kcal**	**Kcal_AF**	**Kalt**	**Kpel**	**Kpau**	**Kvan**	**Kstr**	**Kbor**	**Kviri**
Occurrence points	596	50	235	194	48	25	130	11	14
FC	LQHP	LQH	LQH	LQH	LQHP	LQ	LQHP	L	LQ
RM	1,5	4	1,5	3	3	2,5	2,5	2	1,5
AUC_{TEST}	0,90	0,98	0,84	0,87	0,91	0,90	0,95	0,93	0,94

AUC_{DIFF}	0,01	0,02	0,03	0,02	0,02	0,04	0,01	0,06	0,02
**Predictor**	% contribution								
Annual Mean Temperature (Bio1)	2.91	4,43	12,36	1,85	1,51	0,18	1,59	38,41	5,59
Mean Diurnal Range (Bio2)	0,15	0	3,51	0	0,75	3,78	6,62	49,98	1,48
Isothermality (Bio3)	1,65	0,31	0,47	3,29	0	0	6	0	22,93
Temperature Seasonality (Bio4)	30,91	50,73	19,36	52,29	9,47	10,63	15,2	0	0
Temperature Annual Range (Bio7)	2,79	39,10	0,97	0,89	2,93	0	1,54	0	0
Annual Precipitation (Bio12)	23,77	0	3,79	0,08	23,24	2,13	0,31	0	0
Precipitation Seasonality (Bio15)	9,31	0	0, 26	17,51	2,72	13,84	31,25	5,13	14,91
Precipitation of Driest Quarter (Bio17)	3,15	0	29,62	10,48	10.12	27,84	22,63	0	0
Precipitation of Warmest Quarter (Bio18)	15,33	0	5,05	5,13	5,56	16,85	2,45	0	9,56
Precipitation of Coldest Quarter (Bio19)	4,86	1,02	14,97	0,75	32,40	16,27	0,73	5,75	0,69
Tree-cover	5,18	4,40	9,65	7,72	9,57	8,48	9,72	0,74	2,13
Global aridity index	0	0	0	0	1,72	0	2,15	0	42,70
