# Supplementary material for: The combined role of dispersal and niche evolution in the diversification of Neotropical lizards
Source: Ecol Evol. 2020 Feb 14;10(5):2608–25. doi: 10.1002/ece3.6091 (PMC7069304; doi:10.1002/ece3.6091)
Supplement: Supplementary file 11 [file ECE3-10-2608-s011.pdf]

**SUPPORTING INFORMATION**

**The combined role of dispersal and niche evolution in the diversification of Neotropical lizards**

## SUPPORTING TABLES

**Table S6. Molecular markers used at this study for *Kentropyx* spp**. *H* = number of haplotypes; *Hd* = haplotype diversity; *Pi* = Nucleotide diversity (per site).

| Gene | Length (bp) | N/localities | Pi | H/Hd | Model |
|---|---|---|---|---|---|
| cyt *b* | 534 | 97 | 0,1199 | 0,782 | TPM2uf+G |
| 16S | 524 | 170 | 0,0537 | 0,968 | GTR+I+G |
| SNCAIP | 482 | 184 | 0,0043 | 0,658 | HKY+I |
| DNH3 | 646 | 183 | 0,0091 | 0,815 | K80+I |
| R35 | 448 | 184 | 0,0092 | 0,912 | K80+G |
| RP40 | 387 | 180 | 0,0164 | 0,777 | K80+G |
